# Knowledge, attitude, and acceptance of healthcare workers and the public regarding the COVID-19 vaccine: a cross-sectional study

**DOI:** 10.1186/s12889-021-10987-3

**Published:** 2021-05-20

**Authors:** Muhammed Elhadi, Ahmed Alsoufi, Abdulmueti Alhadi, Amel Hmeida, Entisar Alshareea, Mawadda Dokali, Sanabel Abodabos, Omaymah Alsadiq, Mohammed Abdelkabir, Aimen Ashini, Abdulhamid Shaban, Saja Mohammed, Nehal Alghudban, Eman Bureziza, Qasi Najah, Khawla Abdulrahman, Nora Mshareb, Khawla Derwish, Najwa Shnfier, Rayan Burkan, Marwa Al-Azomi, Ayman Hamdan, Khadeejah Algathafi, Eman Abdulwahed, Khadeejah Alheerish, Naeimah Lindi, Mohamed Anaiba, Abobaker Elbarouni, Monther Alsharif, Kamal Alhaddad, Enas Alwhishi, Muad Aboughuffah, Wesal Aljadidi, Aisha Jaafari, Ala Khaled, Ahmed Zaid, Ahmed Msherghi

**Affiliations:** 1 Faculty of Medicine, University of Tripoli, University Road, Furnaj, 13275 Tripoli, Libya; 2 Faculty of Medicine, University of Zawia, Az Zawiyah, Libya; 3 Faculty of Medicine, Sebha University, Sebha, Libya; 4 Faculty of Medicine, University of Benghazi, Benghazi, Libya; 5 Faculty of Medicine, University of AL-Mergib, Al Khums, Libya; 6 Faculty of Medicine, Al-Jabal Al Gharbi University, Gherian, Libya; 7 Faculty of medicine, Omer Al Mukhtar University, Al Bayda, Libya; 8 Faculty of Medical Technology, University of Tripoli, Tripoli, Libya; 9 Faculty of Medicine, University of Ajdabiya, Ajdabiya, Libya; 10 Faculty of Medicine, Misurata University, Misurata, Libya; 11 Faculty of Dentistry, university of Benghazi, Benghazi, Libya

**Keywords:** COVID-19, SARS-CoV-2, Vaccine, Acceptance, Knowledge, Attitude

## Abstract

**Background:**

This study determined the knowledge, attitudes, and practice regarding COVID-19 and assessed the acceptance of the COVID-19 vaccine among healthcare workers and the general population.

**Methods:**

A web-based, cross-sectional study was conducted using convenience sampling in Libya from December 1 to 18, 2020 among the general population and healthcare workers. Data on demographic characteristics, COVID-19 vaccination-related concerns, knowledge, attitudes, and practice regarding COVID-19, and knowledge, attitudes, and acceptance regarding the COVID-19 vaccine were collected using a self-administered survey. A binomial logistic regression was performed with 70% efficacy to determine the association between acceptance of the vaccine and study variables.

**Results:**

Valid and complete responses were collected from 15,087 participants. Of these, 6227 (41.3%) were male and 8860 (58.7%) were female, with a mean (SD) age of 30.6 ± 9.8 years. Moreover, 485 (3.2%) participants were infected with COVID-19 at the time of the study, while 2000 (13.3%) had been previously infected. Overall, 2452 (16.3%) participants agreed, and 3127 (20.7%) strongly agreed, with “having concerns about serious vaccine-related complications.” Mask-wearing adherence was reported by 10,268 (68.1%) of the participants. Most participants (14,050, 93.1%) believed that the vaccine should be provided for free, while 7272 (48.2%) were willing to buy it. Regarding vaccine acceptance and efficacy, 12,006 (79.6%) reported their willingness to take the vaccine with an efficacy of 90% or more, 9143 (60.6%) with an efficacy of 70% or more, and only 6212 (41.2%) with an efficacy of 50%. The binomial logistic regression revealed that vaccine acceptance was not associated with belonging to the medical field versus the general population. Acceptance was statistically associated with younger age groups, especially 31–40 (OR = 1.3 [1.09, 1.55]) and 41–50 years (OR = 1.29, [1.09, 1.54]). However, having a family member or friend infected with COVID-19 was positively associated with the likelihood of vaccine acceptance (OR = 1.09 [1.02, 1.18]), while having a friend or family member who died due to COVID-19 was negatively associated with it (OR = 0.89 [0.84, 0.97]).

**Conclusions:**

Acceptance of the COVID-19 vaccine is an essential determinant of vaccine uptake and the likelihood of controlling the COVID-19 pandemic. Developing strategies to decrease public hesitation and increase trust is vital for implementing vaccination programs.

**Supplementary Information:**

The online version contains supplementary material available at 10.1186/s12889-021-10987-3.

## Background

The Coronavrus Disease 2019 (COVID-19) pandemic caused severe disruptions in and unprecedented challenges for healthcare systems worldwide. Severe acute respiratory syndrome coronavirus 2 (SARS-CoV-2), causative of severe viral pneumonia that started in Wuhan, China in December 2019, has infected more than 120 million people and resulted in 2.66 million deaths as of March 16, 2021 [1].

COVID-19 primarily affects the respiratory system with a range of symptoms from mild rhinorrhea to severe respiratory distress syndrome [2, 3]. This virus is generally more fatal for the elderly and those with a history of comorbidities, such as hypertension, obesity, diabetes, and kidney disease [4, 5].

African healthcare systems are not well-equipped to tackle this pandemic [6]. While African countries are at a higher risk of disease spread due to limited health infrastructure and training, their inability to promptly obtain the vaccine further increases the risk of disease spread. Not only have many developed countries ordered most of the vaccine supplies, but vaccine-related costs and transfer issues may also further delay vaccination procedures for African people as far as late 2021 or early 2022 [7].

The first case of COVID-19 in Libya was reported on March 24, 2020 [8]. Since then, the pandemic has spread rapidly here, resulting in more than 146,000 cases and 2402 deaths as of March 16, 2021. However, Libya’s healthcare system was not prepared for this pandemic and continues to suffer from several issues such as shortage of personal protective equipment, lack of healthcare training, unavailability of testing centers in many cities, and shortage of healthcare center funding due to the ongoing civil war conflict and financial crisis; these factors have resulted in several unprecedented financial, psychological, and social challenges for healthcare workers [9–11].

For decades, vaccinations have been considered the best method to control rapidly spreading infectious diseases. That said, many groups and individuals recently started to spread rumors and conspiracy theories aimed against vaccination, intensifying the pressure on healthcare authorities and workers [12]. COVID-19 vaccine development and supply is an ongoing process [13]; currently, in Europe and North America, several candidate vaccines from well-known companies have been released for healthcare workers and high-risk populations such as the elderly and patients with chronic diseases [14]. However, low- and middle-income countries are at risk of vaccination delays due to several reasons: lack of public trust, shortage of resources, and scarcity of vaccination supply as many high-income countries secure a large amount of the new vaccines, without prioritizing other countries. Consequently, this inequality can leave low- and middle-income countries at a disadvantage, given their low ability to fight COVID-19 with their current status of healthcare system, leading to humanitarian crises [15]. A new collaboration by several companies and their initiatives announced in September 2020 aimed at supplying 100 million doses of COVID-19 vaccine to low- and middle-income countries in 2021 [16].

To achieve the necessary herd immunity to control viral transmission and stop the pandemic, vaccinating more than 82% of the population is crucial and requires strong acceptance and low hesitation levels throughout the population [17]. Therefore, identifying factors associated with vaccine acceptance and hesitancy is needed to implement policy changes and help public health experts identify a conceptual framework and educational campaign aimed at increasing this awareness in the general population [18].

Waning public confidence in vaccines due to rumors and conspiracy theories is a major challenge for public health experts and policymakers worldwide [19]. Hesitation, spreading rumors, and fake news can affect public mentality and vaccine decisions. A known example is the 2003–2004 Nigerian boycott of the polio vaccine that resulted in a surge of the disease [20, 21]. Therefore, social endorsement and efforts against hesitation regarding the COVID-19 vaccination are essential, especially in limited-resource settings. This will help promote vaccination and establish trust between the general population and health authorities and policymakers, leading to better control of the pandemic and a reduction of lives lost. Therefore, ascertaining vaccine acceptance and hesitation among the general population and healthcare workers is crucial to draw policy plans and assess available resources to meet COVID-19 and overall health challenges to lessen the acute pandemic burden. This study determined the knowledge, attitudes, and practice pertaining to the COVID-19 pandemic. We also examined the COVID-19 vaccine knowledge, attitudes, and acceptance among the general population and healthcare providers.

## Methods

A cross-sectional online survey was conducted involving the general population, medical students, and healthcare workers in more than 20 Libyan cities. The study was conducted between December 1 and 18, 2020.

### Study design, setting, and period

**Fig. 1:**
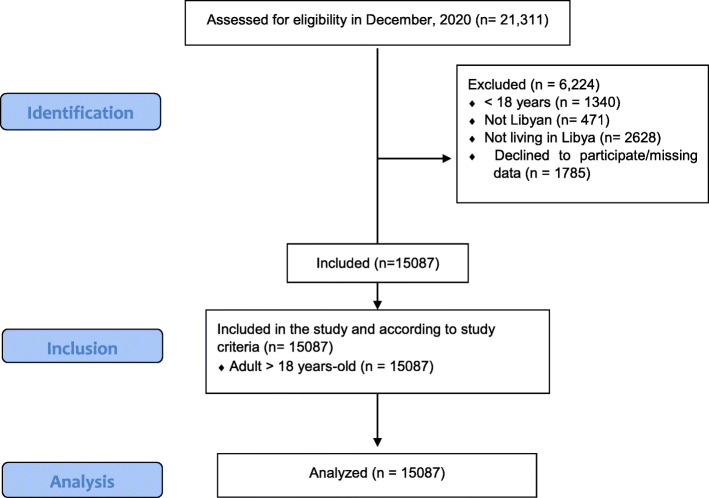
STROBE flow chart. STROBE, Strengthening the Reporting of Observational Studies in Epidemiology

The online survey using Google Forms targeted the general population by sending the survey to a list of emails and social media platforms (e.g., Facebook and WhatsApp) with specific questions about nationality and residency status to avoid selection bias. The survey was also conducted among healthcare workers and medical students through specific social media platforms with questions about employment and educational status to ensure collection of valid samples. The survey was conducted anonymously without identity-related data but with specific questions for the general Libyan population and healthcare workers to ensure the appropriate population selection. The study’s reporting follows the Strengthening the Reporting of Observational Studies in Epidemiology statement [22], as shown in a flow chart in Fig. 1.

### Sample size and sampling technique

According to the United Nations data and Worldometers, the Libyan population was 6,917,632 as of December 31, 2020. To reach participants, convenience sampling and snow ball sampling methods were used. The sample size was calculated based on a single proportion formula, considering a sample proportion of 50% while using a cross-sectional study design wherein *n* = required sample size (*n* = Z (α/2) 2 pq/d2) and 95% CI with 1% margin of error. Therefore, we required a sample size of 9590 as the study’s target population to represent the general population.

### Participants

Only Libyan nationals, or those currently living in Libya, and those aged above 18 years were included. We estimated the number of clicks on the survey link to repesent approximately 21,311 possible participant responses.

### Measures

The survey contained the following sections:

- Sociodemographic section: One page of the first section consisted of study information and an informed consent agreement. This comprised questions related to gender, age, specific nationality, employment status, geographical residency region, marital status, monthly income in Libyan Dinars (LYD), presence of financial difficulties, availability of fixed income, and educational level.

#### - perception of the COVID-19 pandemic and vaccination concerns

This section contained questions on general concerns and attitudes regarding the COVID-19 pandemic, including speculation on the time needed to control a pandemic, confidence in the government, opinion on controlling the COVID-19 pandemic through vaccine usage, shortage and difficulties of children’s vaccines, safety and trust in the COVID-19 vaccine, opinion about difficulties of vaccine distribution, concerns about potential complications from the vaccine, and whether they preferred a specific COVID-19 vaccine.

#### - knowledge, attitude, and practice regarding COVID-19 pandemic

The questionnaire was developed based on a literature review of earlier studies and discussions among authors after several in-depth interviews and advice from public health and epidemiology experts [23, 24]. The final version of the questionnaire had 23 items with 5,8, and 10 items in the knowledge, attitude, and practice sections, respectively. Each correct answer was given a point toward the final score of each section. Supplementary file 1 provides the final version of the survey along with correct answers that were scored.

#### - knowledge, attitude, and acceptance regarding the COVID-19 vaccine

The final version of the vaccine questionnaire was developed based on recent literature reviews of vaccine questionnaire studies and several open-ended interviews conducted by the authors [25–30]. The questionnaire items were edited, with questions added or removed based on qualitative data and structured interviews [31]. The knowledge section contained three items, and the attitude and acceptance sections each contained six items. Each correct survey answer added one point toward the final score of each section. Supplementary file 1 presents the final version with the scoring method.

A score of 70% or more in each section of both surveys was determined as the cut-off score for adequate knowledge, attitude, practice, or acceptance. Both questionnaires were developed in English and tested in a pilot study involving 30 participants. Subsequently, a series of revisions to ensure high internal consistency by Cronbach’s alpha was performed. The initial sample determined for pilot study was not included in the analysis.

The survey was developed in English and was forward-backward translated to Arabic to accommodate the local language. Two independent translators worked on the Arabic version. Along with linguistic and public health experts, we compared the two versions for a final consensus.

The knowledge, attitude, and acceptance regarding COVID-19 vaccine questionnaire had an internal consistency, with Cronbach’s alpha values of 0.797 for the English version and 0.748 for the Arabic version. The knowledge, attitude, and practice regarding COVID-19 questionnaire had a high internal consistency, with Cronbach’s alpha values of 0.771 for the English version and 0.753 for the Arabic version. Both questionnaire and study tools are presented in Supplementary file 1. The online survey was conducted according to the Checklist for Reporting Results of Internet E-Surveys (CHERRIES) [32].

### Statistical analysis

Frequency, percentage, mean, and standard deviation were used in the descriptive statistical analysis. A chi-square test was performed for categorical variables, while the Mann–Whitney U test was performed for continuous variables. Binomial logistic regression was used to determine the impact of study variables on COVID-19 vaccine acceptance. Statistical analysis was performed using IBM’s SPSS Statistics package for Windows (Version 25.0).

### Ethical approval

The Bioethics Committee at the Biotechnology Research Center of the Ministry of Higher Education and Scientific Research in Libya approved the study. All participants provided informed consent prior to their participation.

## Results

### Main study findings

A total of 15,087 respondents were included in the final analysis, of which 6227 (41.3%) were male and 8860 (58.7%) were female, with a mean (SD) age of 30.6 ± 9.8 years, ranging from 18 to 72 years. Among the participants, 11,120 (73.7%) were from the general population, 1752 (11.6%) were medical students, 1394 (9.2%) were medical doctors, and 821 (5.4%) were paramedics or nurses. More than half the respondents, (9036, 59.9%) were married, and most (12,065, 80%) had attained post-secondary education (university or college level of education). Geographically, most respondents (10,678, 70.8%) were from the most populated western Libya. Regarding financial status, 6085 (40.3%) had an income of < LYD1000, with 6514 (43.2%) between LYD1000–2500 (USD 1 is equivalent to LYD 4.46). About half the respondents (6714, 44.5%) had financial difficulties.

Among study participants, 485 (3.2%) were infected with COVID-19 at the time of the study, while 2000 (13.3%) had been previously infected. However, more than half the participants, (8564, 56.8%) reported having a family member or friend infected with COVID-19, and 5189 (34.4%) had lost a friend or family member to COVID-19. Table 1 provides an overview of the study characteristics and differences between the study populations.

**Table 1:** Characteristics of study participants (*n* = 15,087)

Variables	Total (%) *n* = 15,087	General Population (%) *n* = 11,120	Medical Students (%) *n* = 1752	Physicians (%) *n* = 1394	Paramedic (%) *n* = 821	*P*-value
**Age range (years)**						< 0.001*
18–30	8513 (56.4%)	5819 (52.3%)	1695 (96.7%)	531 (38.1%)	468 (57%)	
31–40	4353 (28.9%)	3343 (30.1%)	50 (2.9%)	683 (49%)	277 (33.7%)	
41–50	1546 (10.2%)	1354 (12.2%)	6 (0.3%)	132 (9.5%)	54 (6.6%)	
**>** 50	675 (4.5%)	604 (5.4%)	1(0.1%)	48 (3.4%)	22 (2.7%)	
**Gender**						< 0.001*
Female	8860 (58.7%)	6144 (55.3%)	1244 (71%)	953 (68.4%)	519 (63.2%)	
Male	6227 (41.3%	4976 (44.7%)	508 (29%)	441 (31.6%)	302 (36.8%)	
**Marital status**						
Married	9036 (59.9%)	6386 (57.4%)	1635 (93.3%)	579 (41.5%)	436 (53.1%)	< 0.001*
Not married (Including widow and divorce status)	6051 (40.1%)	4734 (42.6%)	117 (6.7%)	815 (58.5%)	385 (46.9%)	
**Education level**						< 0.001*
Elementary	53 (0.4%)	48 (0.4%)	0 (0%)	0 (0%)	0 (0%)	
Middle school	326 (2.2%)	310 (2.8%)	0 (0%)	0 (0%)	3 (0.4%)	
High school	2643 (17.5%)	2477 (22.3%)	0 (0%)	0 (0%)	72 (8.8%)	
Post-secondary Studies	12,065 (80%)	8285 (74.5%)	1752 (100%)	1394 (100%)	746 (90.9%)	
**Geographical region**						< 0.001*
West	10,678 (70.8%)	7944 (71.4%)	1184 (67.6%)	1018 (73%)	532 (64.8%)	
East	2025 (13.4%)	1412 (12.7%)	348 (19.9%)	176 (12.6%)	89 (10.8%)	
South	676 (4.5%)	487 (4.4%)	67 (3.8%)	51 (3.7%)	71 (8.6%)	
Center	1708 (11.3%)	1277 (11.5%)	153 (8.7%)	149 (10.7%)	129 (15.7%)	
**Monthly Income**						< 0.001*
< 1000 LYD	6085 (40.3%)	4560 (41%)	645 (36.8%)	476 (34.1%)	404 (49.2%)	
1000–2500 LYD	6514 (43.2%)	4724 (42.5%)	770 (43.9%)	686 (49.2%)	334 (40.7%)	
2500–4000 LYD	1680 (11.1%)	1235 (11.1%)	233 (13.3%)	150 (10.8%)	62 (7.6%)	
> 4000 LYD	808 (5.4%)	601 (5.4%)	104 (5.9%)	82 (5.9%)	21 (2.6%)	
**Have financial difficulties**						< 0.001*
Yes	6714 (44.5%)	4948 (44.5%)	716 (10.7%)	627 (45%)	418 (50.9%)	
No	8373 (55.5%)	6167 (55.5%)	1036 (59.1%)	767 (55%)	403 (49.1%)	
**Fixed monthly income**						< 0.001*
Yes	8289 (54.9%)	6297 (56.6%)	538 (30.7%)	932 (66.9%)	522 (63.9%)	
No	6798 (45.1%)	4823 (43.4%)	1214 (69.3%)	462 (33.1%)	299 (36.4%)	
**Currently Infected with COVID-19**						0.344
Yes	485 (3.2%)	369 (3.3%)	46 (2.6%)	48 (3.4%)	22 (2.7%)	
No	14,602 (96.8%)	10,751(96.7%)	1706 (97.4%)	1346(96.6%)	799 (97.3%)	
**Previously infected with COVID-19**						< 0.001*
Yes	2000 (13.3%)	1403 (12.6%)	230 (13.1%)	241 (17.3%)	126 (15.3%)	
No	13,087 (86.7%)	9717 (87.4%)	1522 (86.9%)	1153(82.7%)	695 (84.7%)	
**Have a family member or friend infected with COVID-19?**						< 0.001*
Yes	8564 (56.8%)	6323 (56.9%)	908 (51.8%)	894 (64.1%)	439 (53.5%)	
No	6523 (43.2%)	4797 (43.1%)	844 (48.2%)	500 (35.9%)	382 (46.5%)	
**Have family members or friends died due to COVID-19?**						0.0508
Yes	5189 (34.4%)	3861 (34.7%)	586 (33.4%)	473 (33.9%)	269 (32.8%)	
No	9898 (65.6%)	7259 (65.3%)	1166 (66.6%)	921 (66.1%)	552 (67.2%)	
**The main source of COVID-19 pandemic information?**						< 0.001*
World Health Organization (WHO)	4411 (29.2%)	2970 (26.7%)	628 (35.8%)	564 (40.5%)	249 (30.3%)	
National Center for Disease Control (NCDC)	3873 (25.7%)	2955 (26.6%)	385 (22%)	292 (20.9%)	241 (29.4%)	
News and Media	1576 (10.4%)	1254 (11.3%)	130 (7.4%)	124 (8.9%)	68 (8.3%)	
Internet and Social Media	4771 (31.6%)	3617 (32.5%)	549 (31.3%)	366 (26.3%)	239 (29.1%)	
More than one source	201 (1.3%)	150 (1.3%)	23 (1.3%)	21 (1.5%)	7 (0.9%)	
Other	255 (1.7%)	174 (1.6%)	37 (2.1%)	27 (1.9%)	17 (2.1%)	

*Significant at *P* < 0.001

### Perception of the COVID-19 pandemic and vaccination concerns

Table 2 depicts the findings pertaining to the perception of and concerns about COVID-19 component of the study. Most participants believed that controlling the pandemic would be lengthy, while more than half had confidence in the government’s and healthcare workers’ advice.

**Table 2:** Perception toward the COVID-19 pandemic and vaccination concerns (*n* = 15,087)

Variables	Total (%) *n* = 15,087	General Population (%) *n* = 11,120	Medical Students (%) *n* = 1752	Physicians (%) *n* = 1394	Paramedic (%) *n* = 821	*P*-value
**How long will it take to control the COVID19 pandemic with the current situation and facilities available?**						< 0.001*
2–6 months	1247 (8.3%)	965 (8.7%)	137 (7.8%)	79 (5.7%)	66 (8%)	
4–6 months	2486 (16.5%)	1833 (16.5%)	330 (18.8%)	197 (14.1%)	126 (15.3%)	
6–12 months	4385 (29.1%)	3180 (28.6%)	536 (30.6%)	443 (31.8%)	226 (27.5%)	
More than 12 months	6969 (46.2%)	5142 (46.2%)	749 (24.8%)	675 (48.4%)	403 (49.1%)	
**How confident are you in the advice given by the government and health care providers?**						< 0.001*
Completely confident	5994 (39.7%)	4279 (38.5%)	725 (41.4%)	649 (46.6%)	341 (41.5%)	
Fairly Confident	4866 (32.3%)	3563 (32%)	602 (34.4%)	461 (33.1%)	240 (29.2%)	
Somewhat Confident	3111 (20.6%)	2380 (21.4%)	326 (18.6%)	230 (16.5%)	175 (21.3%)	
Slightly Confident	618 (4.1%)	497 (4.5%)	54 (3.1%)	31 (2.2%)	36 (4.4%)	
Not Confident at all	498 (3.3%)	401 (3.6%)	45 (2.6%)	23 (1.6%)	29 (3.5%)	
**Do you think that the numbers of the reported cases of COVID-19 are being exaggerated?**						< 0.001*
Yes	6018 (39.9%)	4614 (41.5%)	712 (40.6%)	351 (25.2%)	341 (41.5%)	
No	5491 (36.4%)	3747 (33.7%)	667 (38.1%)	789 (56.6%)	288 (35.1%)	
Maybe	3578 (23.7%)	2759 (24.8%)	373 (21.3%)	254 (18.2%)	192 (23.4%)	
**The COVID-19 vaccines, in general, will be useful in controlling the disease.?**						< 0.001*
Strongly agree	4370 (29%)	3326 (29.9%)	398 (22.7%)	402 (28.8%)	244 (29.7%)	
Agree	4015 (26.6%)	2864 (25.8%)	508 (29%)	429 (30.8%)	214 (26.1%)	
Neutral	5040 (33.4%)	3662 (32.9%)	679 (38.8%)	440 (31.6%)	259 (31.5%)	
Disagree	909 (6%)	686 (6.2%)	102 (5.8%)	65 (4.7%)	56 (6.8%)	
Strongly disagree	753 (5%)	582 (5.2%)	65 (3.7%)	58 (4.2%)	48 (5.8%)	
**There are a shortage and difficulty in obtaining children’s vaccines?**						< 0.001*
Yes	8623 (57.2%)	6316 (56.8%)	947 (54.1%)	837 (60%)	523 (63.7%)	
No	1511 (10%)	1114 (10%)	141 (8%)	157 (11.3%)	99 (12.1%)	
Maybe	4953 (32.8%)	3690 (33.2%)	664 (37.9%)	400 (28.7%)	199 (24.2%)	
**Receiving an authorized vaccine for the COVID-19 will be safe and trusty?**						< 0.001*
Strongly agree	3485 (23.1%)	2652 (23.8%)	331 (18.9%)	301 (21.6%)	201 (24.5%)	
Agree	2779 (18.4%)	2003 (18%)	334 (19.1%)	316 (22.7%)	126 (15.3%)	
Neutral	5820 (38.6%)	4209 (37.9%)	750 (42.8%)	545 (39.1%)	316 (38.5%)	
Disagree	1428 (9.5%)	1064 (9.6%)	184 (10.5%)	108 (7.7%)	72 (8.8%)	
Strongly disagree	1575 (10.4%)	1192 (10.7%)	153 (8.7%)	124 (8.9%)	106 (12.9%)	
**There will be difficulty distributing the COVID-19 vaccine equitably and adequately?**						< 0.001*
Yes	10,803 (71.6%)	7845 (70.5%)	1305 (74.5%)	1047 (75.1%)	606 (73.8%)	
No	880 (5.8%)	660 (5.9%)	98 (5.6%)	66 (4.7%)	56 (6.8%)	
Maybe	3404 (22.6%)	2615 (23.5%)	349 (19.9%)	281 (20.2%)	159 (19.4%)	
**In general, I am concerned about serious complications of the vaccines**						< 0.001*
Strongly agree	3127 (20.7%)	2356 (21.2%)	379 (21.6%)	216 (15.5%)	176 (21.4%)	
Agree	2452 (16.3%)	1788 (16.1%)	294 (16.8%)	241 (17.3%)	129 (15.7%)	
Neutral	5348 (35.4%)	3978 (35.8%)	618 (35.3%)	478 (34.3%)	274 (33.4%)	
Disagree	2101 (13.9%)	1509 (13.6%)	260 (14.8%)	214 (15.4%)	118 (14.4%)	
Strongly disagree	2059 (13.6%)	1489 (13.4%)	201 (11.5%)	245 (17.6%)	124 (15.1%)	
**Which of the following COVID-19 vaccine do you prefer to use in the future?**						< 0.001*
Pfizer and BioNTech	596 (4%)	408 (3.7%)	73 (4.2%)	83 (6%)	32 (3.9%)	
Sputnik V	5861 (38.8%)	4162 (37.4%)	682 (38.9%)	683 (49%)	334 (40.7%)	
Oxford/AstraZeneca	1526 (10.1%)	1033 (9.3%)	170 (9.7%)	243 (17.4%)	80 (9.7%)	
None of the above	7104 (47.1%)	5517 (49.6%)	827 (47.2%)	385 (27.6%)	375 (45.7%)	

Some participants (6018, 39.9%) believed that the number of COVID-19 cases was exaggerated, while 4015 (26.6%) agreed, and 4370 (29%) strongly agreed that the COVID-19 vaccine will effectively control the disease, together constituting a majority of participants. However, 8623 (57.2%) reported a shortage of child vaccinations due to disruptions in healthcare services caused by the pandemic.

Regarding the vaccine trust and safety, approximately a third of study participants either agreed (2779, 18.4%) or strongly agreed (3485, 23.1%) that receiving a safe and trusted vaccine was possible. On the other hand, most (10,803, 71.6%) believed there would be difficulties in equitable and proper vaccine distribution. Interestingly, almost a third of the participants either agreed (2452, 16.3%) or strongly agreed (3127, 20.7%) with concerns about serious vaccine complications. A total of 5861 (38.8%) reported their preferences for the Sputnik V vaccine over other candidates. Significant differences between study participant categories were identified, as shown in Table 2.

### Knowledge, attitude, and practice regarding the COVID-19 pandemic

**Fig. 2:**
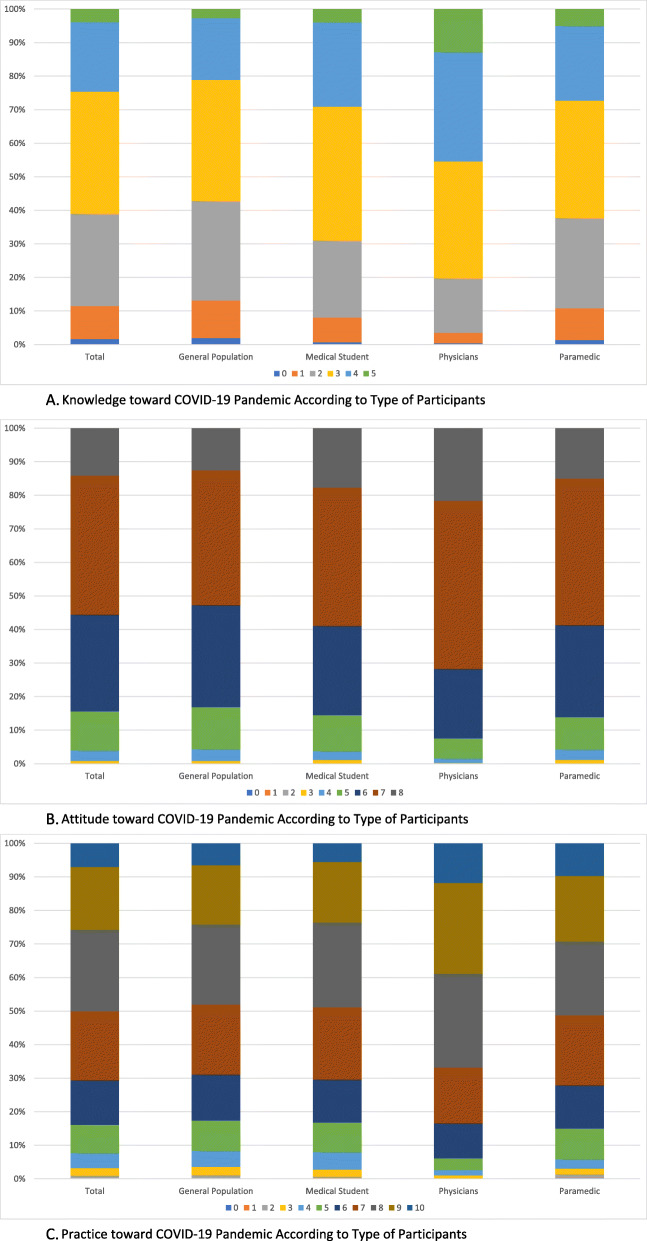
**a** Knowledge toward COVID-19 Pandemic According to Type of Participants. **b**. Attitude toward COVID-19 Pandemic According to Type of Participants. **c**. Practice toward COVID-19 Pandemic According to Type of Participants

The respondents had adequate knowledge of COVID-19, as shown in Table 3. Mean ± SD scores of knowledge, attitude, and practice were 2.7 ± 1.1 (ranging from 0 to 5), 6.5 ± 1.03 (ranging from 2 to 8), and 7.25 ± 1.7 (ranging from 0 to 10), respectively. Figure 2a, b, and c summarize the distribution of knowledge, attitude, and practice scores per participant category.

**Table 3:** Knowledge, Attitude, and Practice toward COVID-19

Questions	Total (%) *n* = 15,087	General Population (%) *n* = 11,120	Medical Students (%) *n* = 1752	Physicians (%) *n* = 1394	Paramedic (%) *n* = 821	***P***-value
***1. Knowledge***						
**1.1 Which of the following liquids is recommended for disinfecting surfaces that have come in contact with COVID-19 patients?**						< 0.001*
**Warm water**	511 (3.4%)	407 (3.7%)	51 (2.9%)	26 (1.9%)	27 (3.3%)	
**25% Alcohol**	893 (5.9%)	735 (6.6%)	76 (4.3%)	41 (2.9%)	41 (5%)	
**70% Alcohol**	7179 (47.6%)	4875 (43.8%)	984 (56.2%)	880 (63.1%)	440 (53.6%)	
**95% Alcohol**	6504 (43.1%)	5103 (45.9%)	641 (36.6%)	447 (32.1%)	313 (38.1%)	
**1.2 The probability of contracting SARS-CoV-2 infection is lower in the case of:**						< 0.001*
**Talking to an infected person with no social distancing**	4127 (27.4%)	3223 (29%)	340 (19.4%)	276 (19.8%)	288 (35.1%)	
**Sleep with an infected person**	392 (2.6%)	312 (2.8%)	34 (1.9%)	25 (1.8%)	21 (2.6%)	
**Online video chat**	10,568 (70%)	7585 (68.2%)	1378 (78.7%)	1093 (78.4%)	512 (62.4%)	
**1.3 Have you ever been taught how to wear and take-off the facemask according to international safety standards?**						< 0.001*
**Yes**	12,072 (80%)	8691 (78.2%)	1475 (84.2%)	1214 (87.1%)	692 (84.3%)	
**No**	3015 (20%)	2429 (21.8%)	277 (15.8%)	180 (12.9%)	129 (15.7%)	
**1.4 Do you think COVID19-positive women are safe to breastfeed their babies?**						< 0.001*
**Yes**	2740 (18.2%)	1767 (15.9%)	285 (16.3%)	509 (36.5%)	179 (21.8%)	
**No**	6600 (43.7%)	4930 (44.3%)	881 (50.3%)	447 (32.1%)	342 (41.7%)	
**I do not know**	5747 (38.1%)	4423 (39.8%)	586 (33.4%)	438 (31.4%)	300 (36.5%)	
**1.5 Do you think COVID-19 is a severe disease that may cause severe complications?**						< 0.001*
**Yes**	9140 (60.6%)	6660 (59.9%)	1017 (58%)	968 (69.4%)	495 (60.3%)	
**No**	1825 (12.1%)	1341 (12.1%)	249 (14.2%)	131 (9.4%)	104 (12.7%)	
**I do not know**	4122 (27.3%)	3119 (28%)	486 (27.7%)	295 (21.2%)	222 (27%)	
***2. Attitude***						
**2.1 The Novel Corona Virus is undoubtedly human-made to implement particular agendas?**						< 0.001*
**Yes**	5836 (38.7%)	4404 (39.6%)	612 (34.9%)	476 (34.1%)	344 (41.9%)	
**No**	3227 (21.4%)	2258 (20.3%)	453 (25.9%)	345 (24.7%)	171 (20.8%)	
**Maybe**	6024 (39.9%)	4458 (40.1%)	687 (39.2%)	573 (41.4%)	306 (37.3%)	
**2.2 Do you think that the local governmental policies would help reduce the spread of the SARS-CoV-2 virus?**						< 0.001*
**Yes**	4430 (29.4%)	3325 (29.9%)	479 (27.3%)	360 (25.8%)	266 (32.4%)	
**No**	10,657 (70.6%)	7795 (70.1%)	1273 (72.7%)	1034 (74.2%)	555 (67.6%)	
**2.3 Do you believe maintaining a social distance from COVID19 suspected and confirmed cases would negatively impact their psychology?**						< 0.001*
**Yes**	6191 (41%)	4456 (40.1%)	811 (46.4%)	574 (41.2%)	350 (42.6%)	
**No**	8896 (59%)	6664 (59.9%)	941 (53.7%)	820 (58.8%)	471 (57.4%)	
**2.4 Do you think you are not at risk of contracting the COVID-19 because your immunity is strong, and you do not need to follow any precautionary measures?**						< 0.001*
**Yes**	1401 (9.3%)	1112 (10%)	141 (8%)	57 (4.1%)	91 (11.1%)	
**No**	13,686 (90.7%)	10,008 (90%)	1611 (92%)	1337 (95.9%)	730 (88.9%)	
**2.5 Do you believe that the traditional remedies (i.e., herbs) may protect from infectious diseases such as the COVID-19?**						< 0.001*
**Yes**	5513 (36.5%)	4293 (38.6%)	588 (33.6%)	329 (23.6%)	303 (36.9%)	
**No**	9574 (63.5%)	6827 (61.4%)	1164 (66.4%)	1065 (76.4%)	518 (63.1%)	
**2.6 Should family members take care of their COVID-19 patients to reduce the risk of transmitting the infection to a single person?**						< 0.001*
**Yes**	3599 (23.9%)	2796 (25.1%)	422 (24.1%)	213 (15.3%)	168 (20.5%)	
**No**	11,488 (76.1%)	8324 (74.9%)	1330 (75.9%)	1181 (84.7%)	653 (79.5%)	
**2.7 To which extent You agree that physical distancing can protect you and your family from contracting COVID-19 disease?**						< 0.001*
**Strongly agree**	9530 (63.2%)	6958 (62.6%)	1031 (58.8%)	1009 (72.4%)	532 (64.8%)	
**Agree**	2781 (18.4%)	2031 (18.3%)	373 (21.3%)	227 (16.3%)	150 (18.3%)	
**Neutral**	1982 (13.1%)	1503 (13.5%)	266 (15.2%)	117 (8.4%)	96 (11.7%)	
**Disagree**	412 (2.7%)	328 (2.9%)	42 (2.4%)	21 (1.5%)	21 (2.6%)	
**Strongly disagree**	382 (2.5%)	300 (2.7%)	40 (2.3%)	20 (1.4%)	22 (2.7%)	
**2.8 Do you think that following precautionary measures on a personal-level would help the community fight against the COVID-19 pandemic?**						0.058
**Yes**	14,527 (96.3%)	10,681 (96.1%)	1693 (96.6%)	1355 (97.2%)	798 (97.2%)	
**No**	560 (3.7%)	439 (3.9%)	59 (3.4%)	39 (2.8%)	23 (2.8%)	
***3. Practice***						
**3.1 In case you have had contact with the COVID-19 case in the last 2 weeks, and you then have felt feverish or shortness of breath, which of the following steps should you do?**						< 0.001*
**Inform NCDC**	2192 (14.5%)	1658 (14.9%)	208 (11.9%)	186 (13.3%)	140 (17.1%)	
**Inform family and friends.**	4416 (29.3%)	3137 (28.2%)	591 (33.7%)	464 (33.3%)	224 (27.3%)	
**Isolate myself**	8479 (56.2%)	6325 (56.9%)	953 (54.4%)	744 (53.4%)	457 (55.7%)	
**3.2 What should you do if you have been exposed to the COVID-19, and you only informed later on?**						< 0.001*
**Isolate yourself and your family**	12,854 (85.2%)	9511 (85.5%)	1525 (87%)	1150 (82.5%)	668 (81.4%)	
**Put on a face mask**	1051 (7%)	734 (6.6%)	109 (6.2%)	119 (8.5%)	89 (10.8%)	
**Leave home only in urgent situations**	1182 (7.8%)	875 (7.9%)	118 (6.7%)	125 (9%)	64 (7.8%)	
**3.3 Which of the following steps should you follow to take care of a family member who has been in contact with a case infected with SARS-CoV-2?**						< 0.001*
**Keep him/ her in an isolated room with all windows closed to prevent the transmission of infection**	3260 (21.6%)	2529 (22.7%)	395 (22.5%)	175 (12.6%)	161 (19.6%)	
**Cleaning his personal items such as bedding and clothes on a daily basis**	1929 (12.8%)	1508 (13.6%)	171 (9.8%)	136 (9.8%)	114 (13.9%)	
**Allowing friends and relatives to visit him/ her but only individually, not in groups**	257 (1.7%)	211 (1.9%)	24 (1.4%)	7 (0.5%)	15 (1.8%)	
**Washing hands with soap and water and use medical gloves while caring for him/her**	9641 (63.9%)	6872 (61.8%)	1162 (66.3%)	1076 (77.2%)	531 (64.7%)	
**3.4 Which of the following measures should be undertaken to deal with the corpse of a patient who died from COVID-19?**						< 0.001*
**Washing and depositing the deceased is considered safe and must be allowed to respect the relatives and friends.**	634 (4.2%)	512 (4.6%)	59 (3.4%)	32 (2.3%)	31 (3.8%)	
**Funerals should not be allowed at all**	11,788 (78.1%)	8683 (78.1%)	1337 (76.3%)	1105 (79.3%)	663 (80.8%)	
**Funerals are only permitted under strict precautionary policies**	2665 (17.7%)	1925 (17.3%)	356 (20.3%)	257 (18.4%)	127 (15.5%)	
**3.5 What is the best method to clean your hands?**						< 0.001*
**Wash hands only with water**	226 (1.5%)	181 (1.6%)	20 (1.1%)	14 (1%)	11 (1.3%)	
**Wash hands with soap and water**	9246 (61.3%)	6723 (60.5%)	1005 (57.4%)	1003 (72%)	515 (62.7%)	
**Wash hands with a disinfectant hand wash**	5615 (37.2%)	4216 (37.9%)	727 (41.5%)	377 (27%)	295 (36%)	
**3.6 How do you greet your colleagues at work or at school?**						< 0.001*
**By shaking hands**	2350 (15.6%)	1789 (16.1%)	362 (20.7%)	99 (7.1%)	100 (12.2%)	
**By Hugging each other**	406 (2.7%)	296 (2.7%)	85 (4.9%)	8 (0.06%)	17 (2.1%)	
**Only verbal greeting**	12,331 (81.7%)	9035 (81.3%)	1305 (74.5%)	1287 (92.3%)	704 (85.7%)	
**3.7 When are you going to cough or sneeze?**						< 0.001*
**I usually Sneeze and cough into my hand palms**	3208 (21.3%)	2464 (22.2%)	331 (18.9%)	228 (16.4%)	185 (22.5%)	
**I usually sneeze and cough into my elbow**	9473 (62.8%)	6769 (60.9%)	1136 (64.8%)	1043 (74.8%)	525 (63.9%)	
**I prevent myself from coughing/sneezing**	811 (5.4%)	615 (5.5%)	120 (6.8%)	42 (3%)	34 (4.1%)	
**Cough / sneeze freely and without covers, because viruses do not live outside the body**	1595 (10.6%)	1272 (11.4%)	165 (9.4%)	81 (5.8%)	77 (9.4%)	
**3.8 Do you practice social distancing, especially when dealing with people who express symptoms of a cold or a fever?**						< 0.001*
**Yes**	13,684 (90.7%)	10,037 (90.3%)	1566 (89.4%)	1321 (94.8%)	760 (92.6%)	
**No**	1403 (9.3%)	1083 (9.7%)	186 (10.6%)	73 (5.2%)	61 (7.4%)	
**3.9 Do you routinely wear a face mask when you go out?**						< 0.001*
**Yes**	10,268 (68.1%)	7306 (65.7%)	1279 (73%)	1115 (80%)	568 (69.2%)	
**No**	4819 (31.9%)	3814 (34.3%)	473 (27%)	279 (20%)	253 (30.8%)	
**3.10 Do you perform the protective measures, including social distancing, to protect yourself from getting the COVID-19?**						< 0.001*
**Yes**	11,658 (77.3%)	8472 (76.2%)	1344 (76.7%)	1186 (85.1%)	656 (79.9%)	
**No**	3429 (22.7%)	2648 (23.8%)	408 (23.3%)	208 (14.9%)	165 (20.1%)

Among participants, 10,568 (70%) knew that using an online chat system reduces the risk of COVID-19 infection, 12,072 (80%) considered using facemasks, and 9140 (60.6%) regarded COVID-19 to be a serious disease.

Regarding attitude, 5836 (38.7%) considered COVID-19 to be man-made; this indicated a rise in conspiracy theories, which was also present in 34.9% of the medical students and 34.1% of the doctors, although lower than the 39.6% of the general population. Among participants, only 10,657 (70.6%) believed that local government policies would help reduce the spread of COVID-19. Most participants (9574, 63.5%) did not consider herbal remedies to be protective against COVID-19, and most either agreed (2781, 18.4%) or strongly agreed (9530, 63.2%) that social distancing is a protective measure against COVID-19.

The respondents were aware of the practical preventive and management steps of COVID-19. While 8479 (56.2%) reported that they would isolate themselves if they showed COVID-19 symptoms, 12,854 (85.2%) reported that they would isolate themselves and their family members in case of COVID-19 exposure, and 9641 (63.9%) were aware of washing hands with soap and water and using medical gloves to care for COVID-19 patients. Most (11,788, 78.1%) believed that funerals should not be permitted. Of all participants, 12,331 (81.7%) engaged in only verbal social interactions without close physical proximity, while 9473 (62.8%) were aware of proper cough etiquette. Mask-wearing adherence was reported by 10,268 (68.1%) of the participants.

### Knowledge, attitude, and acceptance regarding the COVID-19 vaccine

**Fig. 3:**
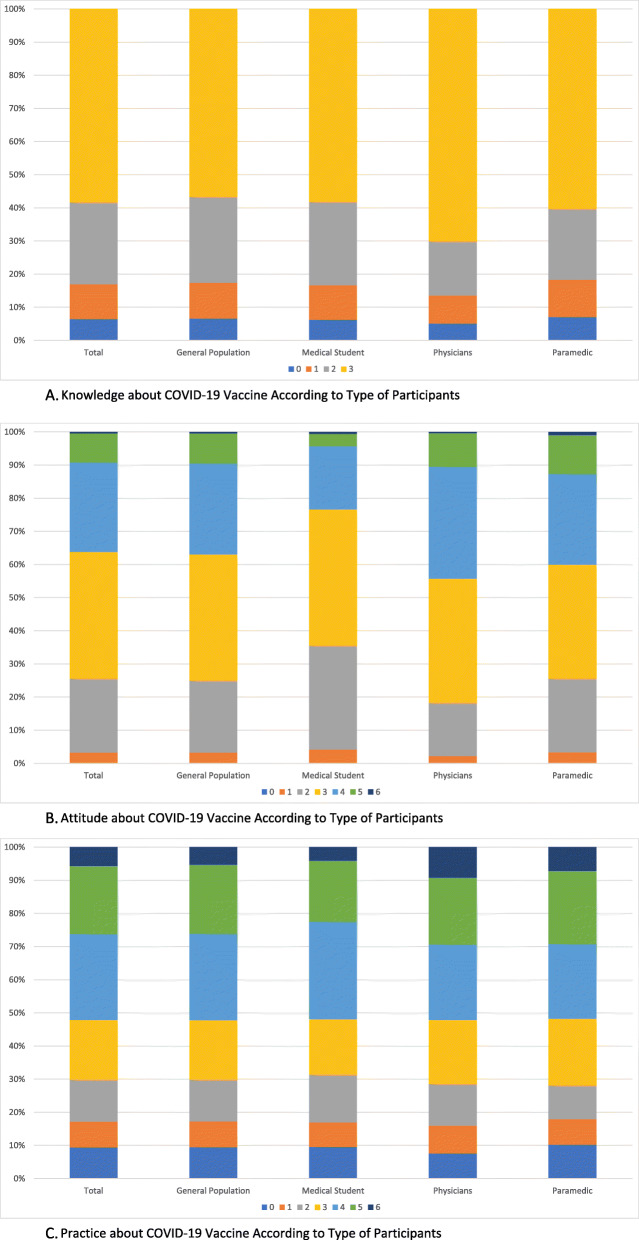
**a**. Knowledge about COVID-19 Vaccine According to Type of Participants. **b** Attitude about COVID-19 Vaccine According to Type of Participants. **c** Practice about COVID-19 Vaccine According to Type of Participants

Overall, the mean ± SD scores for knowledge, attitude, and acceptance were 2.35 ± 0.9 (ranging from 0 to 3), 3.2 ± 0.9 (ranging from 0 to 6), and 3.28 ± 1.7 (ranging from 0 to 6), respectively. Figure 3a, b, and c summarize the distribution of knowledge, attitude, and acceptance scores per participant category. The respondents acknowledged vaccines as essential for children’s health, and 12,970 (86%) believed that vaccination could reduce morbidity and mortality. This number was higher among medical doctors (1220, 87.5%) and students (1528, 87.2%), and slighly lower among the general population (9525, 85.7%). Furthermore, 14,205 (94.2%) believed that finding an effective vaccine was possible and would reduce the COVID-19 burden. However, only 2246 (14.9%) believed that vaccination benefits outweighed the risks. Regarding vaccine purchase and affordability, most participants (14,050, 93.1%) believed that the COVID-19 vaccine should be provided for free, while only 7272 (48.2%) would purchase it if available for sale.

For vaccine acceptance and efficacy, 12,006 (79.6%) reported willingness to take the vaccine with an efficacy of 90% or more, 9143 (60.6%) with an efficacy of 70% or more, and only 6212 (41.2%) with an efficacy of 50%. However, most (12,957, 85.9%) reported that they would encourage their parents to vaccinate. The flu vaccine is an example of vaccine acceptance; only 2040 (13.5%) reported uptake in the last 12 months, and 7082 (46.9%) had planned to take it in the following phase. Table 4 presents the detailed responses to the questionnaire.

**Table 4:** Knowledge, Attitude, and Acceptance of COVID-19 vaccine

Questions	Total (%) *n* = 15,087	General Population (%) *n* = 11,120	Medical Students (%) *n* = 1752	Physicians (%) *n* = 1394	Paramedic (%) *n* = 821	***P***-value
***1. Knowledge***						
**1.1 I think vaccines are important for the health of children?**						0.001
**Yes**	12,584 (83.4%)	9229 (83%)	1467 (83.7%)	1210 (86.8%)	678 (82.6%)	
**No**	875 (5.8%)	690 (6.2%)	81 (4.6%)	58 (4.2%)	46 (5.6%)	
**I do not know / I do not have children**	1628 (10.8%)	1201 (10.8%)	204 (11.6%)	126 (9%)	97 (11.8%)	
**1.2 Being vaccinated against infectious diseases reduces the morbidity and mortality rates of individuals?**						0.002
**Yes**	12,970 (86%)	9525 (85.7%)	1528 (87.2%)	1220 (87.5%)	697 (84.9%)	
**No**	849 (5.6%)	665 (6%)	67 (3.8%)	60 (4.3%)	57 (6.9%)	
**I do not know**	1268 (8.4%)	930 (8.4%)	157 (9%)	114 (8.2%)	67 (8.2%)	
**1.3 Usually, vaccination against infectious diseases is protective and improving the quality of life, especially for people with low immunity and those who suffer from chronic diseases?**						< 0.001*
**Yes**	9881 (65.5%)	7123 (64.1%)	1129 (64.4%)	1075 (77.1%)	554 (67.5%)	
**No**	1572 (10.4%)	1254 (11.3%)	172 (9.8%)	64 (4.6%)	82 (10%)	
**I do not know**	3634 (24.1%)	2743 (24.7%)	451 (25.7%)	255 (18.3%)	185 (22.5%)	
***2. Attitude***						
**2.1 it is possible to find an effective vaccine that could protect against the COVID-19?**						0.002
**Yes**	14,205 (94.2%)	10,470(94.2%)	1670 (95.3%)	1314 (94.3%)	751 (91.5%)	
**No**	882 (5.8%)	650 (5.8%)	82 (4.7%)	80 (5.7%)	70 (8.5%)	
**2.2 If an effective vaccine was found, do you think it could be readily available for everyone?**						< 0.001*
**Yes**	4075 (27%)	3057 (27.5%)	393 (22.4%)	390 (28%)	235 (28.6%)	
**No**	11,012 (73%)	8063 (72.5%)	1359 (77.6%)	1004 (72%)	586 (71.4%)	
**2.3 The benefits of vaccines usually outweigh the risks?**						< 0.001*
**Yes**	2246 (14.9%)	1696 (15.2%)	266 (15.2%)	145 (10.4%)	139 (16.9%)	
**No**	8675 (57.5%)	6315 (56.8%)	971 (55.4%)	923 (66.2%)	466 (56.8%)	
**I do not know**	4166 (27.6%)	3109 (28%)	515 (29.4%)	326 (23.4%)	216 (26.3%)	
**2.4 Do you think the COVID-19 vaccine should be afforded to everyone for free?**						< 0.001*
**Yes**	14,050 (93.1%)	10,355(93.1%)	1616 (92.2%)	1331 (95.5%)	748 (91.1%)	
**No**	1037 (6.9%)	765 (6.9%)	136 (7.8%)	63 (4.5%)	73 (8.9%)	
**2.5 If the COVID-19 vaccine is available for sale, would you buy it?**						0.005
**Yes**	7272 (48.2%)	5344 (48.1%)	820 (46.8%)	709 (50.9%)	399 (48.6%)	
**No**	2456 (16.3%)	1789 (16.1%)	270 (15.4%)	250 (17.9%)	147 (17.9%)	
**Maybe**	5359 (35.5%)	3987 (35.9%)	662 (37.8%)	435 (31.2%)	275 (33.5%)	
**2.6 If you have children, have any of your children ever received a vaccine supposed to protect against diseases that occur during childhood?**						< 0.001*
**Yes**	5965 (39.5%)	4506 (40.5%)	289 (16.5%)	776 (55.7%)	394 (48%)	
**No**	580 (3.8%)	457 (4.1%)	44 (2.5%)	43 (3.1%)	36 (4.4%)	
**I do not have children**	8542 (56.6%)	6157 (55.4%)	1419 (81%)	575 (41.2%)	391 (47.6%)	
***3. Acceptance***						
**3.1 If a COVID-19 vaccine is available with an efficacy of 95%, would you be a candidate for receiving all shots?**						0.204
**Yes**	12,006 (79.6%)	8832 (79.4%)	1393 (79.5%)	1138 (81.6%)	643 (78.3%)	
**No**	3081 (20.4%)	2288 (20.6%)	359 (20.5%)	256 (18.4%)	178 (21.7%)	
**3.2 If a COVID-19 vaccine is available with an efficacy of 70%, would you be a candidate for receiving the vaccine?**						0.171
**Yes**	9143 (60.6%)	6768 (60.9%)	1070 (61.1%)	806 (57.8%)	499 (60.8%)	
**No**	5944 (39.4%)	4352 (39.1%)	682 (38.9%)	588 (42.2%	322 (39.2%)	
**3.3 If a COVID-19 vaccine is available with an efficacy of 50%, would you be a candidate for receiving the vaccine?**						< 0.001*
**Yes**	6212 (41.2%)	4660 (41.9%)	744 (42.5%)	494 (35.4%)	314 (38.2%)	
**No**	8875 (58.8%)	6460 (58.1%)	1008 (57.5%)	900 (64.6%)	507 (61.8%)	
**3.4 If a COVID-19 vaccine was available with the desired efficacy, would you encourage your parents to get the vaccine?**						0.135
**Yes**	12,957 (85.9%)	9542 (85.8%)	1495 (85.3%)	1224 (87.8%)	696 (84.8%)	
**No**	2130 (14.1%)	1578 (14.2%)	257 (14.7%)	170 (12.2%)	125 (15.2%)	
**3.5 Did you receive the seasonal flu shot in the last 12 months?**						< 0.001*
**Yes**	2040 (13.5%)	1412 (12.7%)	199 (11.4%)	290 (20.8%)	139 (16.9%)	
**No**	13,047 (86.5%)	9708 (87.3%)	1553 (88.6%)	1104 (79.2%)	682 (83.1%)	
**3.6 Are you planning to receive a seasonal flu vaccine in the next year?**						< 0.001*
**Yes**	7082 (46.9%)	5161 (46.4%)	718 (41%)	769 (55.2%)	434 (52.9%)	
**No**	8005 (53.1%)	5959 (53.6%)	1034 (59%)	625 (44.8%)	387 (47.1%)	

A univariate chi-square test and multivariate binomial logistic regression were performed to determine the association between acceptance of the COVID-19 vaccine and study variables, as shown in Table 5. For univariate analysis, only marital status, geographical region, whether currently infected with COVID-19, having a family member infected with COVID-19, and having family members or friends who died due to COVID-19 were statistically associated with acceptance of a vaccine with an efficacy of 70% or more; *p* < 0.05.

**Table 5:** Association between acceptance to receive COVID-19 vaccine with 70% efficacy and study characteristics

Variables	Total (%) *n* = 15,087	Accept to receive COVID-19 vaccine	Do not accept to receive COVID-19 vaccine	*p*-value	Odds Ratio	95% CI for Odds Ration		*P*-value
						Lower	Upper	
**Population Characteristic**	15,087	9143 (60.6%)	5944 (39.4%)	0.171				
General population	11,120 (73.7%)	6768 (74%)	4352 (73.2%)		1.00 (ref)			0.14
Medical students	1752 (11.6%)	1070 (11.7%)	682 (11.5%		1.03	0.88	1.19	0.71
Physician	1394 (9.2%)	806 (8.8%)	588 (9.9%)		1.07	0.89	1.27	0.45
Paramedic	821 (5.4%)	499 (5.5%)	322 (5.4%)		0.91	0.76	1.08	0.27
**Age range (years)**				0.060				
18–30	8513 (56.4%)	5159 (56.4%)	3354 (56.4%)		1.00 (ref)			0.01*
31–40	4353 (28.9%)	2678 (29.3%)	1675 (28.2%)		1.3	1.09	1.55	0.003*
41–50	1546 (10.2%)	927 (10.1%)	619 (10.4%)		1.29	1.09	1.54	0.002*
**>** 50	675 (4.5%)	379 (4.1%)	296 (5%)		1.18	0.98	1.42	0.08*
**Gender**				0.349				
Female	8860 (58.7%)	5397 (59%)	3463 (58.3%)		1.00 (ref)			
Male	6227 (41.3%	3746 (41%)	2481 (41.7%)		1.04	0.97	1.12	0.23
**Marital status**				0.032*				
Married	9036 (59.9%)	3730 (40.8%)	2321 (39%)		1.00 (ref)			
Not married (Including widow and divorce status)	6051 (40.1%)	5413 (59.2%)	3623 (61%)		0.85	0.78	0.93	< 0.001**
**Education level**				0.337				
Elementary	53 (0.4%)	30 (0.3%)	18 (0.3)		1.00 (ref)			0.32
Middle school	326 (2.2%)	191 (2.1%)	122 (2.1%)		1.09	0.61	1.98	0.76
High school	2643 (17.5%)	1585 (17.3%)	964 (16.2%)		1.03	0.81	1.29	0.83
Post-secondary Studies	12,065 (80%)	7337 (80.2%)	4840 (81.4%)		1.09	0.99	1.2	0.06
**Geographical region**				< 0.001**				
West	10,678 (70.8%)	6346 (69.4%)	4332 (72.9%)		1.00 (ref)			< 0.001**
East	2025 (13.4%)	1280 (14%)	745 (12.5%)		0.86	0.78	0.96	0.009
South	676 (4.5%)	438 (4.8%)	238 (40%)		1.01	0.88	1.15	0.85
Center	1708 (11.3%)	1079 (11.8%)	629 (10.6%)	0.032*	1.07	0.89	1.29	0.45
**Monthly Income**								
< 1000 LYD	6085 (40.3%)	3768 (41.2%)	2317 (39%)		1.00 (ref)			0.19
1000–2500 LYD	6514 (43.2%)	3900 (42.7%)	2614 (44%)		1.02	0.87	1.18	0.81
2500–4000 LYD	1680 (11.1%)	984 (10.8%)	696 (11.7%)		0.95	0.82	1.11	0.56
> 4000 LYD	808 (5.4%)	491 (5.4%)	317 (5.3%)	0.38	0.91	0.77	1.08	0.31
**Have financial difficulties**								
Yes	6714 (44.5%)	4095 (44.8%)	2619 (44.1%)		1.01	0.94	1.08	0.87
No	8373 (55.5%)	5048 (55.2%)	3325 (55.9%)	0.28	1.00 (ref)			
**Fixed monthly income**								
Yes	8289 (54.9%)	4991 (54.6%)	3298 (55.5%)		1.03	0.96	1.11	0.41
No	6798 (45.1%)	4125 (45.4%)	2646 (44.5%)	< 0.001*	1.00 (ref)			
**Currently Infected with COVID-19**								
Yes	485 (3.2%)	339 (3.7%)	146 (2.5%)		0.65	0.53	0.79	< 0.001**
No	14,602 (96.8%)	8804 (96.3%)	5798 (97.5%)	0.732	1.00 (ref)			
**Previously infected with COVID-19**								
Yes	2000 (13.3%)	1219 (13.3%)	781 (13.1%)		1.01	0.9	1.11	0.97
No	13,087 (86.7%)	7924 (86.7%)	5163 (86.9%)		1.00 (ref)			
**Have a family member or friend infected with COVID-19?**				0.04*				
Yes	8564 (56.8%)	5130 (56.1%)	3434 (57.8%)		1.09	1.02	1.18	0.01*
No	6523 (43.2%)	4013 (43.9%)	2510 (42.2%)		1.00 (ref)			
**Have a family member or friends died due to COVID-19?**				0.024*				
Yes	5189 (34.4%)	3209 (35.1%)	1980 (33.3%)		0.89	0.84	0.97	0.004*
No	9898 (65.6%)	5934 (64.9%)	3964 (66.7%)		1.00 (ref)			

*Significant at *P* < 0.05

**Significant at *P* < 0.001

For the binomial logistic regression model, medical field or general population affiliation was not associated with acceptance. Acceptance was statistically associated with younger age groups, especially 31–40 years (OR = 1.3 [1.09, 1.55]) and 41–50 years (OR = 1.29, [1.09, 1.54]). However, having a family member or friend infected with COVID-19 was positively associated with the likelihood of vaccine acceptance (OR = 1.09 [1.02, 1.18]), while having a friend or family member who died due to COVID-19 was negatively associated with it (OR = 0.89 [0.84, 0.97]). Interestingly, with other multivariate logistic regression models, being infected with COVID-19 at the time of the study was negatively associated with vaccine acceptance (OR = 0.65, [0.53, 0.79]), while previous contraction of COVID-19 was not statistically associated with COVID-19 vaccine acceptance. There was no statistical association between acceptance of COVID-19 vaccine and gender, monthly income, having financial difficulty, having a fixed income, and being previously infected with COVID-19.

## Discussion

Availability and efficacy of the COVID-19 vaccine are vital to successfully control the pandemic. Policymakers and health authorities must ensure acceptance and trust from both the community and healthcare workers because hesitation and delay may result in vaccination refusal. This could lead to devastating effects in public health and hinder the healthcare system’s ability to accommodate the challenges of the pandemic. Our study provided an overview of the acceptance and knowledge of the COVID-19 vaccine by Libyan healthcare workers and the general population.

In this nationwide study, we found an adequate level of knowledge, attitude, and acceptance regarding COVID-19 vaccinations. Approximately, 60.6% of the study population were willing to receive the COVID-19 vaccine with an efficacy of 70% or more and 79.6% with an efficacy of 90%. However, we did not find a statistically significant difference among the general population, medical students, medical doctors, and paramedics. According to our results, the general public in Libya had a clear understanding of COVID-19 and a favourable attitude toward it. However, we discovered some issues in the public’s understanding of COVID-19 and their actions in response to it. Approximately half of the participants (56.2%) were aware that isolation of themselves if they have COVID-19 symptoms, putting them at risk of disease exposure. Mask-wearing adherence was reported among 68.1%, while 18.4% strongly agreed, and 63.2% agreed, to social distancing as protective measures, indicating social compliance with established guidelines of physical distancing. However, this is less than previously reported in China where 96.6% of the general public adhered to wearing facemasks [33].

Although medical doctors and students showed higher acceptance of the COVID-19 vaccination, this was not statistically different among the general population, medical students, and healthcare providers such as doctors and nurses. This indicates that all populations prefer vaccination, implying a general willingness to take the vaccination even though acceptance was proportionally related to vaccine efficacy.

Our study found that 71.6% believed COVID-19 vaccine distribution would be difficult, given the circumstances and challenges in Libya. We also found that 20.7% strongly agreed, and 16.3% agreed, with having concerns about possible severe complications from the vaccine. That said, doctors ranked the lowest for concerns on vaccine complications wherein 15.5% strongly agreed, and 17.3% agreed, to having such concerns, which may trigger COVID-19 vaccine hesitation. Stronger public health expert interventions and large-scale population-based campaigns are needed to reduce such hesitation and build public trust on this issue.

A global survey regarding COVID-19 vaccine acceptance in 19 countries with 13,426 respondents found that acceptance varies between countries and income level, with China having 90% and Russia, 55% potential public acceptance of the vaccine [27]. They found that higher-income participants were highly likely to accept the vaccine. However, our study did not find a statistically significant difference between acceptance and income level. Lazarus et al. [27] reported a high level of trust in government recommendations, slightly similar to our findings wherein 39.7% were completely confident, and 32.3% fairly confident, in the government and healthcare providers’ advice. Another study in April 2020 on 911 US adults found that 57.6% were willing to be vaccinated, while another study in May 2020 involving 5000 US participants found that 31.1% did not intend to be vaccinated. In a recent study published in December 2020 of 1878 US individuals found that 52% were very likely, and 27% somewhat likely, to receive COVID-19 vaccinations, while 7% would not take the vaccine [34].

A study conducted in Indonesia found that 93.3% would accept a vaccine with an efficacy of 95%, while 67% would do so with a 50% efficacy, which is higher than our findings wherein 79.6% would accept the COVID-19 vaccine with a 95% efficacy and 60.6% with a 50% efficacy [30]. Another study conducted involving the Saudi Arabian general population found that 64.7% were willing to take the vaccine [35]. The latest systemic review of recent literature on the general populations of 33 countries found that vaccination acceptance varies based on geographical locations and income levels. There is low acceptance in countries such as Kuwait (23.6%) and Jordan (28.4%), moderate to half acceptance in countries such as Italy (53.7%), Poland (56.3%), and Russia (54.9%). By contrast, some countries exhibited high acceptance, especially in east Asia, such as Indonesia (93.3%), China (91.3%), and Malaysia (94.3%). This suggests that vaccine acceptance should be encouraged and increased to achieve the population-based immunity needed to control the pandemic.

Only 14.9% of our study participants reported that vaccine benefits outweighed the risks, and only 48.2% reported their willingness to buy the vaccine if available for sale. Concerns and hesitation regarding COVID-19 vaccination safety as well as public trust issues may hinder vaccination intake among the general public and healthcare workers. Therefore, public immunization programs and educational campaigns about the vaccine’s importance should be designed to increase public trust, remove financial and social barriers, alleviate the public health issues, and boost trust and vaccine intake.

Interestingly, our study revealed 38.7% of the participants believed conspiracy theories that COVID-19 is a man-made viral disease. The general population showed a higher percentage in this belief (39.6%) than doctors (34.1%). However, despite doctors possessing greater knowledge about the virus, they still believe in this conspiracy theory.

Therefore, the government and public health experts must take the necessary measures according to the local culture to achieve higher vaccination acceptance and encourage positive intention toward COVID-19 vaccination. An educational framework must also be produced for the general population conveying the risks of vaccine delay or avoidance as it can then reduce governmental efforts to control the pandemic. Ultimately, a transparent educational and social campaign portraying social benefits of vaccination is critical to alleviate the detrimental pandemic effects [36, 37].

This study provides insightful information on educational awareness about COVID-19 infection and vaccination that can be implemented via an applicable framework of governmental public health efforts. Health literacy and awareness greatly influence intention to act upon health recommendations, which is crucial to avoid such negative consequences of the pandemic, such as waste disposal of protective gear and restrictive hygienic practices aimed to reduce the COVID-19 public health burden [38, 39]. Therefore, building health literacy through a social and educational framework is needed to prepare individuals for difficult situations such as pandemics to be socially responsible and to assure successful vaccination campaigns among the general population [40].

Based on our knowledge, this is the first study conducted in the African region, which is still suffering from the consequences of the first COVID-19 wave due to an unprepared healthcare system. This resulted in high mortality and complications among the African population, recording the highest intensive care mortality in the world (48.2% mortality rate after 30 days critical care admission) [41]. African countries, such as Libya, suffer from a shortage of personal protective equipment, low availability of mechanical ventilators, a lack of training for healthcare providers, high psychological and mental stress, and a scarcity of governmental support for COVID-19 facilities [6, 42].

This study has the following strengths. First, it collected detailed and complete data with a large sample size and 15,087 responses. Second, two tools assessed knowledge, attitudes, and practices pertaining to the COVID-19 pandemic as well as knowledge, attitudes, and acceptance regarding COVID-19 vaccines, adequately covering the general population, medical students, and healthcare workers such as doctors and paramedics. It also compared the responses to identify differences. Finally, it provided an initial report from the African region involving a country within a limited setting. Thus, it provides valuable data for policymakers to plan vaccination programs and tackle the challenges identified in the study.

The study has some limitations. First, the cross-sectional survey method may not able to draw a conclusion and strong association; thus, there is further longitudinal studies are needed. Second, online survey distribution may have missed people from older age groups or specific lower socioeconomic classes that may not have access to the Internet. This might potentially impact the generalizability of the results. Third, the study was conducted in African countries with specific circumstances, limiting the results’ international generalizability. However, we obtained similar findings to recently published studies from other countries. Finally, perceived acceptance based on the survey may not reveal true acceptance of the vaccine in reality. Thus, interpreting results to actuality must be done cautiously.

## Conclusion

Our study demonstrated the knowledge, attitudes, and practices pertaining to the COVID-19 pandemic and COVID-19 vaccine-related knowledge, attitudes, and acceptance in the Libyan population during the ongoing pandemic. The current study was able to provide a thorough review of Libyans’ understanding, attitudes, and practices regarding COVID-19. According to the results, Libyans have an adequate degree of knowledge and awareness about COVID-19 and are generally optimistic about resolving the pandemic. Addressing the public concerns, raising awareness about COVID-19 vaccination as a disease-control method, addressing conspiracy theories, reducing hesitation toward vaccination, and increasing efforts toward to provide vaccines in countries with limited resources, such as Libya and other African regions, to prevent further deterioration of general public health due to COVID-19 is imperative.

## Supplementary Information

**Additional file 1.**

## Data Availability

The datasets used and/or analyzed during the current study are available from the corresponding author upon reasonable request.

## Declaration

All methods were carried out in accordance with relevant guidelines and regulations.

## References

[1] Dong E, Du H, Gardner L (2020). An interactive web-based dashboard to track COVID-19 in real time. Lancet Infect Dis.

[2] Guan WJ, Ni ZY, Hu Y, Liang WH, Ou CQ, He JX, Liu L, Shan H, Lei CL, Hui DSC, du B, Li LJ, Zeng G, Yuen KY, Chen RC, Tang CL, Wang T, Chen PY, Xiang J, Li SY, Wang JL, Liang ZJ, Peng YX, Wei L, Liu Y, Hu YH, Peng P, Wang JM, Liu JY, Chen Z, Li G, Zheng ZJ, Qiu SQ, Luo J, Ye CJ, Zhu SY, Zhong NS, China Medical Treatment Expert Group for Covid-19 (2020). Clinical characteristics of coronavirus disease 2019 in China. N Engl J Med.

[3] Huang C, Wang Y, Li X, Ren L, Zhao J, Hu Y, Zhang L, Fan G, Xu J, Gu X (2020). Clinical features of patients infected with 2019 Novel coronavirus in Wuhan, China. Lancet.

[4] Bhatraju PK, Ghassemieh BJ, Nichols M, Kim R, Jerome KR, Nalla AK, Greninger AL, Pipavath S, Wurfel MM, Evans L, Kritek PA, West TE, Luks A, Gerbino A, Dale CR, Goldman JD, O’Mahony S, Mikacenic C (2020). Covid-19 in critically ill patients in the Seattle region - case series. N Engl J Med.

[5] Grasselli G, Zangrillo A, Zanella A, Antonelli M, Cabrini L, Castelli A, Cereda D, Coluccello A, Foti G, Fumagalli R, Iotti G, Latronico N, Lorini L, Merler S, Natalini G, Piatti A, Ranieri MV, Scandroglio AM, Storti E, Cecconi M, Pesenti A, Agosteo E, Alaimo V, Albano G, Albertin A, Alborghetti A, Aldegheri G, Antonini B, Barbara E, Belgiorno N, Belliato M, Benini A, Beretta E, Bianciardi L, Bonazzi S, Borelli M, Boselli E, Bronzini N, Capra C, Carnevale L, Casella G, Castelli G, Catena E, Cattaneo S, Chiumello D, Cirri S, Citerio G, Colombo S, Coppini D, Corona A, Cortellazzi P, Costantini E, Covello RD, de Filippi G, Dei Poli M, Della Mura F, Evasi G, Fernandez-Olmos R, Forastieri Molinari A, Galletti M, Gallioli G, Gemma M, Gnesin P, Grazioli L, Greco S, Gritti P, Grosso P, Guatteri L, Guzzon D, Harizay F, Keim R, Landoni G, Langer T, Lombardo A, Malara A, Malpetti E, Marino F, Marino G, Mazzoni MG, Merli G, Micucci A, Mojoli F, Muttini S, Nailescu A, Panigada M, Perazzo P, Perego GB, Petrucci N, Pezzi A, Protti A, Radrizzani D, Raimondi M, Ranucci M, Rasulo F, Riccio M, Rona R, Roscitano C, Ruggeri P, Sala A, Sala G, Salvi L, Sebastiano P, Severgnini P, Sforzini I, Sigurtà FD, Subert M, Tagliabue P, Troiano C, Valsecchi R, Viola U, Vitale G, Zambon M, Zoia E, COVID-19 Lombardy ICU Network (2020). Baseline characteristics and outcomes of 1591 patients infected with SARS-CoV-2 admitted to ICUs of the Lombardy region, Italy. JAMA.

[6] Elhadi M, Msherghi A, Alkeelani M, Alsuyihili A, Khaled A, Buzreg A, Boughididah T, Abukhashem M, Alhashimi A, Khel S, Gaffaz R, Ben Saleim N, Bahroun S, Elharb A, Eisay M, Alnafati N, Almiqlash B, Biala M, Alghanai E (2020). Concerns for low-resource countries, with under-prepared intensive care units, facing the COVID-19 pandemic. Infect Dis Health.

[7] More than 85 poor countries will not have widespread access to coronavirus vaccines before 2023. The-Economist 2021, https://www.eiu.com/n/85-poor-countries-will-not-have-access-to-coronavirus-vaccines/. Accessed 18 Dec 2020.

[8] Elhadi M, Momen AA, Ali Senussi Abdulhadi OM. A COVID-19 case in Libya acquired in Saudi Arabia. Travel Med Infect Dis 2020:101705. 10.1016/j.tmaid.2020.101705. 10.1016/j.tmaid.2020.101705 PMC7252057 32360409

[9] Elhadi M, Msherghi A, Alkeelani M, Zorgani A, Zaid A, Alsuyihili A, Buzreg A, Ahmed H, Elhadi A, Khaled A (2020). Assessment of Healthcare Workers? Levels of Preparedness and Awareness Regarding COVID-19 Infection in Low-Resource Settings. Am J Trop Med Hyg.

[10] Elhadi M, Msherghi A, Elgzairi M, Alhashimi A, Bouhuwaish A, Biala M, Abuelmeda S, Khel S, Khaled A, Alsoufi A, Elmabrouk A, Alshiteewi FB, Alhadi B, Alhaddad S, Gaffaz R, Elmabrouk O, Hamed TB, Alameen H, Zaid A, Elhadi A, Albakoush A (2020). Psychological status of healthcare workers during the civil war and COVID-19 pandemic: a cross-sectional study. J Psychosom Res.

[11] Elhadi M, Msherghi A, Elgzairi M, et al. Burnout Syndrome Among Hospital Healthcare Workers During the COVID-19 Pandemic and Civil War: A Cross-Sectional Study. 2020;11:579563. 10.3389/fpsyt.2020.579563. 10.3389/fpsyt.2020.579563 PMC7759513 33362600

[12] Paterson P, Meurice F, Stanberry LR, Glismann S, Rosenthal SL, Larson HJ (2016). Vaccine hesitancy and healthcare providers. Vaccine.

[13] Lurie N, Saville M, Hatchett R, Halton J (2020). Developing Covid-19 vaccines at pandemic speed. N Engl J Med.

[14] Voysey M, Clemens SAC, Madhi SA, Weckx LY, Folegatti PM, Aley PK, et al. Safety and efficacy of the ChAdOx1 nCoV-19 vaccine (AZD1222) against SARS-CoV-2: an interim analysis of four randomised controlled trials in Brazil, South Africa, and the UK. Lancet. 2021;397(10269):99–111. 10.1016/S0140-6736(20)32661-1. 10.1016/S0140-6736(20)32661-1 PMC7723445 33306989

[15] Elhadi M, Msherghi A, Alkeelani M, et al. Concerns for low-resource countries, with under-prepared intensive care units, facing the COVID-19 pandemic. Infect Dis Health. 2020;25(4):227–32. 10.1016/j.idh.2020.05.008. 10.1016/j.idh.2020.05.008 PMC7274573 32631682

[16] Alliance GtV (2020). New collaboration makes further 100 million doses of COVID-19 vaccine available to low- and middle-income countries.

[17] Sanche S, Lin YT, Xu C, Romero-Severson E, Hengartner N, Ke R (2020). High contagiousness and rapid spread of severe acute respiratory syndrome coronavirus 2. Emerg Infect Dis.

[18] Wong MCS, Wong ELY, Huang J, Cheung AWL, Law K, Chong MKC, Ng RWY, Lai CKC, Boon SS, Lau JTF, Chen Z, Chan PKS (2021). Acceptance of the COVID-19 vaccine based on the health belief model: a population-based survey in Hong Kong. Vaccine.

[19] Kumar D, Chandra R, Mathur M, Samdariya S, Kapoor N (2016). Vaccine hesitancy: understanding better to address better. Israel J Health Policy Res.

[20] Ghinai I, Willott C, Dadari I, Larson HJ (2013). Listening to the rumours: what the northern Nigeria polio vaccine boycott can tell us ten years on. Glob Public Health.

[21] Heymann DL, Sutter RW, Aylward RB (2006). Polio eradication: interrupting transmission, towards a polio-free world.

[22] von Elm E, Altman DG, Egger M, Pocock SJ, Gotzsche PC, Vandenbroucke JP (2008). The strengthening the reporting of observational studies in epidemiology (STROBE) statement: guidelines for reporting observational studies. J Clin Epidemiol.

[23] Zhong B-L, Luo W, Li H-M, Zhang Q-Q, Liu X-G, Li W-T, Li Y (2020). Knowledge, attitudes, and practices towards COVID-19 among Chinese residents during the rapid rise period of the COVID-19 outbreak: a quick online cross-sectional survey. Int J Biol Sci.

[24] Ferdous MZ, Islam MS, Sikder MT, Mosaddek ASM, Zegarra-Valdivia JA, Gozal D (2020). Knowledge, attitude, and practice regarding COVID-19 outbreak in Bangladesh: an online-based cross-sectional study. Plos One.

[25] de Figueiredo A, Simas C, Karafillakis E, Paterson P, Larson HJ (2020). Mapping global trends in vaccine confidence and investigating barriers to vaccine uptake: a large-scale retrospective temporal modelling study. Lancet.

[26] Malik AA, McFadden SM, Elharake J, Omer SB (2020). Determinants of COVID-19 vaccine acceptance in the US. EClinicalMed.

[27] Lazarus JV, Ratzan SC, Palayew A, et al. A global survey of potential acceptance of a COVID-19 vaccine. Nat Med. 2021;27:225–8. 10.1038/s41591-020-1124-9. 10.1038/s41591-020-1124-9 PMC7573523 33082575

[28] Muqattash R, Niankara I, Traoret RI (2020). Survey data for COVID-19 vaccine preference analysis in the United Arab Emirates. Data Brief.

[29] Biasio LR, Bonaccorsi G, Lorini C, Pecorelli S. Assessing COVID-19 vaccine literacy: a preliminary online survey. Hum Vaccin Immunother. 2021;17(5):1304–12. 10.1080/21645515.2020.1829315. 10.1080/21645515.2020.1829315 PMC8078752 33118868

[30] Harapan H, Wagner AL, Yufika A, Winardi W, Anwar S, Gan AK, Setiawan AM, Rajamoorthy Y, Sofyan H, Mudatsir M. Acceptance of a COVID-19 Vaccine in Southeast Asia: A Cross-Sectional Study in Indonesia. Frontiers Public Health. 2020;8:381. 10.3389/fpubh.2020.00381. 10.3389/fpubh.2020.00381 PMC7372105 32760691

[31] Ricci L, Lanfranchi JB, Lemetayer F, Rotonda C, Guillemin F, Coste J, Spitz E (2019). Qualitative methods used to generate questionnaire items: a systematic review. Qual Health Res.

[32] Eysenbach G (2004). Improving the quality of web surveys: the checklist for reporting results of internet E-surveys (CHERRIES). J Med Internet Res.

[33] Cheng VC-C, Wong S-C, Chuang VW-M, So SY-C, Chen JH-K, Sridhar S, Chan JF-W, Hung IF-N, Ho P-L, To KK-W (2020). The role of community-wide wearing of face mask for control of coronavirus disease 2019 (COVID-19) epidemic due to SARS-CoV-2. J Inf Secur.

[34] Khubchandani J, Sharma S, Price JH, Wiblishauser MJ, Sharma M, Webb FJ (2021). COVID-19 vaccination hesitancy in the United States: a rapid national assessment. J Community Health.

[35] Al-Mohaithef M, Padhi BK (2020). Determinants of COVID-19 vaccine acceptance in Saudi Arabia: a web-based National Survey. J Multidiscip Healthc.

[36] García LY, Cerda AA (2020). Acceptance of a COVID-19 vaccine: a multifactorial consideration. Vaccine.

[37] Harrison EA, Wu JW (2020). Vaccine confidence in the time of COVID-19. Eur J Epidemiol.

[38] Islam SMD, Safiq MB, Bodrud-Doza M, Mamun MA. Perception and Attitudes Toward PPE-Related Waste Disposal Amid COVID-19 in Bangladesh: An Exploratory Study. Front Public Health. 2020;8:592345. 10.3389/fpubh.2020.592345. 10.3389/fpubh.2020.592345 PMC7691663 33304879

[39] Islam SMD-U, Mondal PK, Ojong N, Bodrud-Doza M, Siddique MAB, Hossain M, Mamun MA: Water, sanitation, hygiene and waste disposal practices as COVID-19 response strategy: insights from Bangladesh. Environ Dev Sustain. 2021:1–22. 10.1007/s10668-020-01151-9. 10.1007/s10668-020-01151-9 PMC7778416 33424423

[40] Paakkari L, Okan O (2020). COVID-19: health literacy is an underestimated problem. Lancet Public Health.

[41] Investigators TA: An African, multi-Centre evaluation of patient care and clinical outcomes for patients with COVID-19 infection admitted to high-care or intensive care units. Lancet. 2021, Accepted in Press. 10.1016/S0140-6736(21)00441-4 PMC8137309 34022988

[42] Tabah A, Ramanan M, Laupland KB, Buetti N, Cortegiani A, Mellinghoff J, Conway Morris A, Camporota L, Zappella N, Elhadi M, Povoa P, Amrein K, Vidal G, Derde L, Bassetti M, Francois G, Ssi Yan Kai N, de Waele JJ, PPE-SAFE contributors (2020). Personal protective equipment and intensive care unit healthcare worker safety in the COVID-19 era (PPE-SAFE): an international survey. J Crit Care.

