# Supplementary material for: Knowledge, attitude, and acceptance of healthcare workers and the public regarding the COVID-19 vaccine: a cross-sectional study
Source: BMC Public Health. 2021 May 20;21:955. doi: 10.1186/s12889-021-10987-3 (PMC8136114; doi:10.1186/s12889-021-10987-3)
Supplement: Supplementary file 1 — Additional file 1. [file 12889_2021_10987_MOESM1_ESM.docx]

**Knowledge, Attitude, and Acceptance of Healthcare Workers and the Public Regarding the COVID-19 Vaccine: A Cross-sectional Study**

**Muhammed Elhadi**^1*^**, Ahmed Alsoufi^1^, Abdulmueti Alhadi^2^, Amel Hmeida^1^, Entisar Alshareea^1^, Mawadda Dokali^1^, Sanabel Abodabos^1^, Omaymah Alsadiq^2^ , Mohammed Abdelkabir^3^ , Aimen Ashini^1^, Abdulhamid Shaban^1^ , Saja Mohammed^2^, Nehal Alghudban^2^, Eman Bureziza^4^, Qasi Najah^5^, Khawla Abdulrahman^3^, Nora Mshareb^1^, Khawla Derwish^1^, Najwa Shnfier^1^, Rayan Burkan^1^, Marwa Al-Azomi^2^, Ayman Hamdan^6^ , Khadeejah Algathafi^7^ , Eman Abdulwahed^6^, Khadeejah Alheerish^3^, Naeimah lindi^7^, Mohamed Anaiba^8^, Abobaker Elbarouni^8^, Monther Alsharif^9^, Kamal Alhaddad^1^, Enas Alwhishi^1^, Muad Aboughuffah^5^, Wesal Aljadidi^1^ , Aisha jaafari^1^, Ala Khaled^1^, Ahmed Zaid^1^, Ahmed Msherghi^1^**

^1^ *Faculty of Medicine, University of Tripoli, Tripoli, Libya*

^2^ *Faculty of Medicine, University of Zawia, Az Zawiyah, Libya.*

^3^ *Faculty of Medicine, Sebha University, Sebha, Libya.*

^4^ *Faculty of Medicine, University of Benghazi, Benghazi, Libya*

^5^ *Faculty of Medicine, University of AL-Mergib, Al Khums, Libya*

^6^ *Faculty of Medicine, Al-Jabal Al Gharbi University, Gherian, Libya.*

^7^ *Faculty of Medicine, University of Ajdabiya, Ajdabiya, Libya.*

^6^ *Faculty of Medical Technology, University of Tripoli, Tripoli, Libya*

^7^ *Faculty of medicine, Omer Al Mukhtar University, Libya*

^8^ *Faculty of Medicine, Misurata University, Misurata, Libya*

^9^ *Faculty of Dentistry, university of Benghazi, Benghazi, Libya*

* **Correspondence: Dr. Muhammed Elhadi**, Faculty of Medicine, University of Tripoli, University Road, Furnaj, 13275, Tripoli, Libya (Email: Muhammed.elhadi.uot@gmail.com) (Telephone: +218945196407)

**Supplementary File 1**

English version of the study questionnaire.

1. **Age range (years)**

**___________________**

1. **Your Gender:**

Male

Female

1. **Marital Status:**

Not married (Including widow and divorce status)

Married

1. **Your Educational level:**

Elementary school

Middle School

High School

Post-secondary education (i.e. Bachelor’s Degree)

Post-graduate Degree

1. **Geographical region**

The East

The Middle

The East

The South

1. **Monthly income**

< than 1000 LYD

1000-2500LYD

2500-4000 LYD

> 4000 LYD

1. **Have financial difficulties**

Yes

No

1. **Fixed monthly income**

Yes

No

1. **Currently Infected with COVID-19**

Yes

No

1. **Previously infected with COVID-19**

Yes

No

1. **Have a family member or friend infected with COVID-19?**

Yes

No

1. **Have family members or friends died due to COVID-19?**

Yes

No

1. **The main source of COVID-19 pandemic information?**

World Health Organization (WHO)

National Center for Disease Control (NCDC)

News and Media

Internet and Social Media

More than one source

Other

1. **How long will it take to control the COVID19 pandemic with the current situation and facilities available?**

2-6 months

4-6 months

6-12 months

More than 12 months

1. **How confident are you in the advice given by the government and health care providers?**

Completely confident

Fairly Confident

Somewhat Confident

Slightly Confident

Not Confident at all

1. **Do you think that the numbers of the reported cases of COVID-19 are being exaggerated?**

Yes

No

Maybe

1. **The COVID-19 vaccines, in general, will be useful in controlling the disease.?**

Strongly agree

Agree

Neutral

Disagree

Strongly disagree

1. **There are a shortage and difficulty in obtaining children's vaccines?**

Yes

No

Maybe

1. **Receiving an authorized vaccine for the COVID-19 will be safe and trusty?**

Strongly agree

Agree

Neutral

Disagree

Strongly disagree

1. **There will be difficulty distributing the COVID-19 vaccine equitably and adequately?**

Yes

No

Maybe

1. **In general, I am concerned about serious complications of the vaccines**

**Strongly agree**

Agree

Neutral

Disagree

Strongly disagree

1. **Which of the following COVID-19 vaccine do you prefer to use in the future?**

Pfizer and BioNTech

Sputnik V

Oxford/AstraZeneca

None of the above

TAB 3

**1.1 Which of the following liquids is recommended for disinfecting surfaces that have come in contact with COVID-19 patients?**

Warm water

25% Alcohol

70% Alcohol

95% Alcohol

**1.2 The probability of contracting SARS-CoV-2 infection is lower in the case of:**

Talking to an infected person with no social distancing

Sleep with an infected person

Online video chat

**1.3 Have you ever been taught how to wear and take-off the facemask according to international safety standards?**

Yes

No

**1.4 Do you think COVID19-positive women are safe to breastfeed their babies?**

Yes

No

I do not know

**1.5 Do you think COVID-19 is a severe disease that may cause severe complications?**

Yes

No

I do not know

**2.1 The Novel Corona Virus is undoubtedly human-made to implement particular agendas?**

Yes

No

Maybe

**2.2 Do you think that the local governmental policies would help reduce the spread of the SARS-CoV-2 virus?**

Yes

No

**2.3 Do you believe maintaining a social distance from COVID19 suspected and confirmed cases would negatively impact their psychology?**

Yes

No

**2.4 Do you think you are not at risk of contracting the COVID-19 because your immunity is strong, and you do not need to follow any precautionary measures?**

Yes

No

**2.5 Do you believe that the traditional remedies (i.e., herbs) may protect from infectious diseases such as the COVID-19?**

Yes

No

**2.6 Should family members take care of their COVID-19 patients to reduce the risk of transmitting the infection to a single person?**

Yes

No

**2.7 To which extent You agree that physical distancing can protect you and your family from contracting COVID-19 disease?**

Strongly agree

Agree

Neutral

Disagree

Strongly disagree

**2.8 Do you think that following precautionary measures on a personal-level would help the community fight against the COVID-19 pandemic?**

Yes

No

**3.1 In case you have had contact with the COVID-19 case in the last two weeks, and you then have felt feverish or shortness of breath, which of the following steps should you do?**

Inform NCDC

Inform family and friends.

Isolate myself

**3.2 What should you do if you have been exposed to the COVID-19, and you only informed later on?**

Isolate yourself and your family

Put on a face mask

Leave home only in urgent situations

**3.3 Which of the following steps should you follow to take care of a family member who has been in contact with a case infected with SARS-CoV-2?**

Keep him/ her in an isolated room with all windows closed to prevent the transmission of infection

Cleaning his personal items such as bedding and clothes on a daily basis

Allowing friends and relatives to visit him/ her but only individually, not in groups

Washing hands with soap and water and use medical gloves while caring for him/her

**3.4 Which of the following measures should be undertaken to deal with the corpse of a patient who died from COVID-19?**

Washing and depositing the deceased is considered safe and must be allowed to respect the relatives and friends.

Funerals should not be allowed at all

Funerals are only permitted under strict precautionary policies

**3.5 What is the best method to clean your hands?**

Wash hands only with water

Wash hands with soap and water

Wash hands with a disinfectant hand wash

**3.6 How do you greet your colleagues at work or at school?**

By shaking hands

By Hugging each other

Only verbal greeting

**3.7 When are you going to cough or sneeze?**

I usually Sneeze and cough into my hand palms

I usually sneeze and cough into my elbow

I prevent myself from coughing/sneezing

Cough / sneeze freely and without covers, because viruses do not live outside the body

**3.8 Do you practice social distancing, especially when dealing with people who express symptoms of a cold or a fever?**

Yes

No

**3.9 Do you routinely wear a face mask when you go out?**

Yes

No

**3.10 Do you perform the protective measures, including social distancing, to protect yourself from getting the COVID-19?**

Yes

No

**4.1 I think vaccines are important for the health of children?**

Yes

No

I do not know / I do not have children

**4.2 Being vaccinated against infectious diseases reduces the morbidity and mortality rates of individuals?**

Yes

No

I do not know

**4.3 Usually, vaccination against infectious diseases is protective and improving the quality of life, especially for people with low immunity and those who suffer from chronic diseases?**

Yes

No

I do not know

**4.1 it is possible to find an effective vaccine that could protect against the COVID-19?**

Yes

No

**4.2 If an effective vaccine was found, do you think it could be readily available for everyone?**

Yes

No

**4.3 The benefits of vaccines usually outweigh the risks?**

Yes

No

I do not know

**4.4 Do you think the COVID-19 vaccine should be afforded to everyone for free?**

Yes

No

**4.5 If the COVID-19 vaccine is available for sale, would you buy it?**

Yes

No

Maybe

**4.6 If you have children, have any of your children ever received a vaccine supposed to protect against diseases that occur during childhood?**

Yes

No

I do not have children

**5.1 If a COVID-19 vaccine is available with an efficacy of 95%, would you be a candidate for receiving all shots?**

Yes

No

**5.2 If a COVID-19 vaccine is available with an efficacy of 70%, would you be a candidate for receiving the vaccine?**

Yes

No

**5.3 If a COVID-19 vaccine is available with an efficacy of 50%, would you be a candidate for receiving the vaccine?**

Yes

No

**5.4 If a COVID-19 vaccine was available with the desired efficacy, would you encourage your parents to get the vaccine?**

Yes

No

**5.5 Did you receive the seasonal flu shot in the last 12 months?**

Yes

No

**5.6 Are you planning to receive a seasonal flu vaccine in the next year?**

Yes

No

Arabic version of the study questionnaire

1. العمر ______________
2. الجنس:

(ذكر – انثى)

1. الحالة الاجتماعية:

اعزب

متزوج

1. المستوي التعليمي:

ابتدائي

اعدادي

ثانوي

تعليم جامعي

دراسات عليا

1. الموقع الجغرافي:

غرب طرابلس

شرق طرابلس

جنوب طرابلس

وسط طرابلس

1. الدخل الشهري للأسرة:

اقل من 1000

1000د.ل الى 2500 د.ل

من 2500 د.ل الى 4000 د.ل

اكثر من 4000 د.ل

1. هل تعاني من مشاكل مادية حاليا؟

نعم لا

1. هل تملك دخل شهري ثابت

نعم لا

1. ھل اﻧت ﻣﺻﺎب ﺣﺎﻟﯾﺎ بمرض فيروس كورونا (كوفيد-19)؟

نعم لا

1. هل أصبت سابقا بمرض فيروس كورونا (كوفيد-19)؟

نعم لا

1. هل اصيب احد افراد عائلتك او اصدقائك بكوفيد 19؟

نعم لا

1. هل توفي احد اقاربك او اصدقائك نتيجة اصابته بعدوى كوفيد 19؟

نعم لا

1. ﻣﺎھﻲ ﻣﺻﺎدر ﻣﻌﻠوﻣﺎﺗك اﻻﺳﺎﺳﯾﺔ ﺣول ﻓﺎﯾروس ﻛوروﻧﺎ اﻟﻣﺳﺗﺟد؟

ﻣﻧظﻣﺔ اﻟﺻﺣﺔ اﻟﻌﺎﻟﻣﯾﺔ

اﻟﻣرﻛز اﻟوطﻧﻲ ﻟﻣﻛﺎﻓﺣﺔ اﻻﻣراض

اﻷﺧﺑﺎر واﻟﻘﻧوات اﻻﻋﻼﻣﯾﺔ او اﻟﻌﺎﻟﻣﯾﮫ

اﻻﻧﺗرﻧت ووﺳﺎﺋل اﻟﺗواﺻل اﻻﺟﺗﻣﺎﻋﻲ

اكثر من مصدر من المصادر المذكورة

اخرى

1. ﻣﻊ اﻻﻣﻛﺎﻧﯾﺎت اﻟﻣﺗﺎﺣﺔ ﺣﺎﻟﯾﺎ ,ﻛم ﺗﻌﺗﻘد اﻟﻔﺗرة اﻟﻼزﻣﺔ ﻟﻠﺳﯾطرة ﻋﻠﻰ وﺑﺎء ﻛوروﻧﺎ؟

2-4 اشهر

4-6 اشهر

6-12 شهر

اكثر من 12 شهر

1. ﻣﺎ ﻣدي ﺛﻘﺗك ﻓﻲ اﻟﻧﺻﺎﺋﺢ اﻟﻣﻘدﻣﺔ ﻣن ﻣﻘدﻣﯾن اﻟرﻋﺎﯾﮫ اﻟﺻﺣﯾﮫ ﻣﺗل اﻻطﺑﺎء و اﻟﻣﻣرﺿﯾن؟

واثق تماما واثق متوسط غير واثق غير واثق تمام

1. ھل ﺗﻌﺗﻘد ﺑﺎﻧﮫ ﯾﺗم اﻟﻣﺑﺎﻟﻐﮫ ﻓﻲ اﻻﺻﺎﺑﺎت اﻟﻣﻌﻠﻧﮫ ﺑﻔﺎﯾروس ﻛوروﻧﺎ المستجد ؟

نعم لا ربما

1. ھل ﺗﻌﺗﻘد اﻧﮫ ﻣن اﻟﻣﻣﻛن اﯾﺟﺎد ﻟﻘﺎح ﻓﻌﺎل ﻟﻠﺣﻣﺎﯾﮫ ﻣن ﻓﺎﯾروس ﻛوروﻧﺎ؟

نعم لا

1. اعتقد ان اللقاحات المناعية ﻣﮭﻣﮫ ﻟﺻﺣﺔ اﻻطﻔﺎل

نعم لا لا اعلم

1. أعتقد ان الحصول علي اللقاح المعتمد الخاص بفيروس كورونا (كوفيد-19) سيكون امن وسليم

اوافق بشدة اوافق محايد لااوافق لا اوافق وبشدة

1. ھل ﺗﻌﺗﻘد ﺑﺎﻧﮫ ﺳﺗﻛون ھﻧﺎك ﺻﻌوﺑﺔ ﻓﻲ ﺗوزﯾﻊ ﻟﻘﺎح اﻟﺗطﻌﯾم ﺿد ﻓﺎﯾروس ﻛوروﻧﺎ ﺑﺷﻛل ﻋﺎدل وﺳﻠﯾم ؟

نعم لا ربما

1. اﻧﺎ ﻗﻠق ﺑﺷﺄن اﻟﻣﺿﺎﻋﻔﺎت اﻟﺧطﯾرة ﻟﻠﺗطﻌﯾﻣﺎت ﺑﺷﻛل ﻋﺎم؟

اوافق بشدة اوافق محايد لااوافق لا اوافق وبشدة

1. اي من لقاحات كورونا التالية تفضل استخدامها مستقبلا

اللقاح الروسي SPUTNIK

اللقاح الامريكي الالماني PFIZER AND BIOTECH "فرايزر–بيوتيك"

لقاح شركة موديرنا

لقاح اكسفورد وشركة استرازينيكا

لا شيء مما سبق

1. أي ﻣن اﻟﺳواﺋل اﻻﺗﯾﺔ ﯾﻧﺻﺢ ﺑﮫ ﻟﺗﻌﻘﯾم اﻻﺳطﺢ اﻟﺗﻲ ﻻﻣﺳت ﻣرﯾض ﻣﺻﺎب ﺑﻔﯾروس ﻛوروﻧﺎ اﻟﻣﺳﺗﺟد:

اﻟﻣﺎء اﻟداﻓﺊ

اﻟﻛﺣول ﺗرﻛﯾز 25%

اﻟﻛﺣول ﺗرﻛﯾز75%

اﻟﻛﺣول ﺗرﻛﯾز95%

1. -يعتبر احتمال الاصابة بعدوي مرض فيروس كورونا (كوفيد-19)المستجد أقل في حالة :

تحدثت إﻟﻲ اﻟﻣرﯾض دون وﺟود ﻣﺳﺎﻓﺔ ﻓﺎﺻﻠﺔ

النوم بجانب مريض

تناول الطعام مع مريض باستخدام نفس أدوات الأكل

المحادثة عن طريق البث المباشر

1. هل قام احد من ذوي الاختصاص بتعليمك على الطريقة الصحيحة لارتداء ونزع الكمامة وفقا لمعايير السلامة العالمية؟

نعم لا

1. ھل ﺗﻌﺗﻘد أﻧﮫ ﯾﺳﻣﺢ ﻟﻠﻣرﺿﻌﺔ اﻟﻣﺻﺎﺑﺔ ﺑﻔﯾروس ﻛوروﻧﺎ اﻟﻣﺳﺗﺟد أن ﺗﻘوم ﺑﺎﻛﻣﺎل رﺿﺎﻋﺔ طﻔﻠﮭﺎ

نعم لا ربما

1. ﻓﺎﯾروس ﻛوروﻧﺎ المستجد هو بلا شك من ﺻﻧﻊ اﻟﺑﺷر ﻟﺗﻧﻔﯾذ اﺟﻧدات ﻣﻌﯾﻧة

نعم لا ربما

1. ھل ﺗﻌﺗﻘد أن ﺟﮭود اﻟﺣﻛوﻣﺔ اﻟﻣﺣﻠﯾﺔ ﺳﺗﺳﺎﻋد ﻓﻲ اﻟﺣد ﻣن اﻧﺗﺷﺎر اﻟﻔﯾروس؟

نعم لا

1. ھل ﺗﻌﺗﻘد أن اﻟﻣﺣﺎﻓظﺔ ﻋﻠﻲ ﻣﺳﺎﻓﺔ أﻣﻧﺔ ﻣن اﻟﺣﺎﻻت اﻟﻣﺻﺎﺑﺔ أو اﻟﻣﺧﺎﻟطﺔ ﯾؤﺛر ﺳﻠﺑﺎ ﻋﻠﻲ اﻟﺣﺎﻟﺔ اﻟﻧﻔﺳﯾﺔ ﻟﻠﻣرﯾض / اﻟﻣﺧﺎﻟط

نعم لا

1. هل تعتقد ﺑﺄﻧك ﻏﯾر ﻣﻌرض ﻟﻺﺻﺎﺑﺔ ﺑﻔﯾروس ﻛوروﻧﺎ اﻟﻣﺳﺗﺟد ﻷن ﻣﻧﺎﻋﺗك ﻗوﯾﺔ وﻟﺳت ﺑﺣﺎﺟﺔ ﻻﺗﺑﺎع أي إﺟراءات وﻗﺎﺋﯾﺔ

نعم لا

1. ھل ﺗﻌﺗﻘد أن اﻟﻌﻼﺟﺎت اﻟﺷﻌﺑﯾﺔ اﻟﻣوﺟودة ﻓﻲ ﻣﺟﺗﻣﻌﻧﺎ" كالأعشاب " ﻗد ﺗﺣﻣﻲ ﻣن اﻷﻣراض اﻟﻣﻌدﯾﺔ ﻣﺛل ﻓﯾروس ﻛوروﻧﺎ اﻟﻣﺳﺗجد

نعم لا

1. ھل ﺗﻌﺗﻘد أﻧﮫ ﯾﺟب ﻋﻠﻲ أﻓراد اﻷﺳرة اﻟﺗﻧﺎوب ﻓﻲ رﻋﺎﯾﺔ اﻟﻔرد .اﻟﻣﺻﺎب ﺑﺎﻟﻔﯾروس لتقليل خطر اصابة الافراد الذين يقومون برعاية المريض؟

نعم لا

1. اﻟﻰ أي ﻣدى ﺗواﻓق ان اﻟﺗﺑﺎﻋد اﻻﺟﺗﻣﺎﻋﻲ ﯾﻣﻛن أي ﯾﺣﻣﯾك وﻋﺎﺋﻠﺗك ﻣن اﻻﺻﺎﺑﺔ ﺑﺎﻟﻔﯾروس

اوافق بشدة اوافق محايد لااوافق لا اوافق وبشدة

1. ھل ﺗﻌﺗﻘد أن اﺗﺑﺎع إﺟراءات اﻟﺳﻼﻣﺔ ﻋﻠﻲ اﻟﺻﻌﯾد اﻟﺷﺧﺻﻲ ﺳﯾﻔﯾد اﻟﻣﺟﺗﻣﻊ ﻓﻲ ﻣﻛﺎﻓﺣﺔ ﺟﺎﺋﺣﺔ ﻛوروﻧﺎ

نعم لا

1. في ﺣﺎﻟﺔ ﻣﺧﺎﻟطﺗك ﻟﺣﺎﻟﺔ ﻻ ﺗظﮭر ﻋﻠﯾﮭﺎ اﻷﻋراض ﺧﻼل اﻷﺳﺑوﻋﯾن اﻟﻣﺎﺿﯾﯾن ,ﺛم ﺗﺑﯾن ﻋن طرﯾق اﻟﺗﺣﻠﯾل أن اﻟﻣرﯾض ﻣﺻﺎب ﺑﻔﯾروس * ﻛوروﻧﺎ اﻟﻣﺳﺗﺟد .ﻣﺎ اﻟذي ﯾﺟب ﻓﻌﻠﮫ ﻓﻲ ﻧظرك

اﻟذھﺎب ﻟﻠﻣﺳﺗﺷﻔﻲ

اﻻﺗﺻﺎل ﺑﺎﻟطﺑﯾب ﻣﻊ اﻟﺑﻘﺎء ﻓﻲ اﻟﻣﻧزل

ﺳﺗﻘوم ﺑﻌزل ﻧﻔﺳك

1. إذا ﺧﺎﻟطت ﺷﺧﺻﺎ ﻣﺻﺎﺑﺎ ﺑﻔﯾروس ﻛوروﻧﺎ اﻟﻣﺳﺗﺟد دون ﻋﻠﻣك ,ﺛم ﺗﺑﯾن ﻟك ذﻟك ﻓﻲ اﻟﯾوم اﻟﺗﺎﻟﻲ ,ﻣﺎ اﻟذي ﯾﻧﺑﻐﻲ اﻟﻘﯾﺎم ﺑﮫ ﻓﻲ ﻧظرك؟

عزل ﻧﻔﺳك وأﻓراد أﺳرﺗك

ارﺗداء اﻟﻛﻣﺎﻣﺔ اﻟطﺑﯾﺔ

اﻟﺧروج ﻣن اﻟﻣﻧزل ﻟﻸﺳﺑﺎب اﻟﺿرورﯾﺔ ﻓﻘط

1. اي من الخطوات التالية ينبغي عليك اتباعها للعناية بأحد افراد عائلتك قد كان مخالطا لحالة مصابة بعدوى مرض فيروس كورونا (كوفيد-19)؟

إﺑﻘﺎء اﻟﻣﺧﺎﻟط ﻓﻲ ﻏرﻓﺔ ﻣﻔﺻوﻟﺔ ﻣﻊ اﻏﻼق اﻟﻧواﻓذ وﻗﻧوات اﻟﺗﮭوﯾﺔ ﻟﻣﻧﻊ اﻧﺗﺷﺎر اﻟﻔﯾروس

تنظيف اغراضه الشخصية كفرش السرير وملابسه بشكل يومي

اﻟﺳﻣﺎح ﺑزﯾﺎرة اﻟﻣﺧﺎﻟط وﻟﻛن ﺑﺷﻛل ﻓردي وﻟﯾس ﻛﻣﺟﻣوﻋﺔ

غسل اﻟﯾدﯾن ﺑﺎﻟﻣﺎء واﻟﺻﺎﺑون ﺣﺗﻲ وإن ﻛﻧت ﺗﺳﺗﺧدم اﻟﻘﻔﺎزات اﻟطﺑﯾﺔ أﺛﻧﺎء اﻟﻌﻧﺎﯾﺔ ﺑﺎﻟﺣﺎﻟﺔ

1. أي ﻣن اﻹﺟراءات اﻷﺗﯾﺔ ﯾﻧﺑﻐﻲ اﺗﺑﺎﻋﮭﺎ ﻓﻲ ﻧظرك ﻟﻠﺗﻌﺎﻣل ﻣﻊ ﺟﺛﻣﺎن ﻣرﯾض ﺗوﻓﻲ ﺟراء اﻹﺻﺎﺑﺔ ﺑﻔﯾروس ﻛوروﻧﺎ اﻟﻣﺳﺗﺟد؟

تغسيل اﻟﻣﯾت وﺗودﯾﻌﮫ ﯾﻌﺗﺑر آﻣﻧﺎ وﯾﺟب اﻟﺳﻣﺎح ﺑﮫ ﻟﻣراﻋﺎة أﻗﺎرب اﻟﻣﯾت

ﻻ ﯾﻧﺑﻐﻲ اﻟﺳﻣﺎح ﺑﺗﺷﯾﯾﻊ ﺟﻧﺎزة اﻟﻣﯾ

ﯾﺳﻣﺢ ﺑﺗﺷﯾﯾﻊ اﻟﺟﻧﺎزة وﻟﻛن ﯾﻧﺑﻐﻲ اﺗﺑﺎع إﺟراءات اﻟﺳﻼﻣﺔ

1. ﻛﯾف ﺗﻘوم ﺑﺗﻌﻘﯾم ﯾدﯾك ﻓﻲ اﻟﻌﺎدة

غسبل اﻟﯾدﯾن ﺑﺎﻟﻣﺎء ﻓﻘط

ﻏﺳل اﻟﯾدﯾن ﺑﺎﻟﻣﺎء واﻟﺻﺎﺑون

ﻏﺳل اﻟﯾدﯾن ﺑﺎﺳﺗﺧدام ﻏﺳول ﻣﻌﻘم ﻟﻠﯾدﯾن

1. ﻛﯾف ﺗﻘوم ﺑﺗﺣﯾﺔ زﻣﻼﺋك ﻓﻲ اﻟﻌﻣل أو اﻟدراﺳﺔ؟

ﻋن طرﯾق اﻟﻣﺻﺎﻓﺣﺔ

ﺑﺎﻷﺣﺿﺎن والمعانقة

اﻟﻘﺎء اﻟﺗﺣﯾﺔ ﻋن ﺑﻌد

1. ﻣﺎ ھﻲ اﻟطرﯾﻘﺔ اﻟﺗﻲ ﺗﺗﺑﻌﮭﺎ ﻓﻲ اﻟﺳﻌﺎل واﻟﻌطس؟

اﻟﻌطس ﻓﻲ راﺣﺔ ﯾدﯾك

اﻟﻌطس ﻓﻲ ﻛم اﻟﻘﻣﯾص

أﻣﻧﻊ ﻧﻔﺳﻲ ﻣن اﻟﺳﻌﺎل / اﻟﻌطس

أسعل/ أﻋطس ﻓﻲ اﻟﮭواء اﻟطﻠق ﻷن اﻟﻔﯾروﺳﺎت ﻻ ﺗﻌﯾش ﺧﺎرج اﻟﺟﺳم

1. ھل ﺗﻘوم ﺑﺗطﺑﯾق ﻣﻌﺎﯾﯾر اﻟﻣﺳﺎﻓﺔ اﻻﻣﻧﺔ ﺧﺎﺻﺔ ﻋﻧد اﻟﺗﻌﺎﻣل ﻣﻊ أﺷﺧﺎص ﺗظﮭر ﻋﻠﯾﮭم أﻋراض اﻟزﻛﺎم او اﻟﺣﻣﻰ ؟

نعم لا

1. ھل ﺗﻘوم ﺑﺎﺳﺗﺧدام اﻟﻛﻣﺎﻣﺔ ﺑﺷﻛل ﻣﺳﺗﻣر ﻋﻧد اﻟﺧروج ﻣن اﻟﻣﻧزل؟

نعم لا

1. ھل تقوم ﺑﺎﺟراءات اﻟوﻗﺎﯾﺔ واﻟﺗﺑﺎﻋد اﻻﺟﺗﻣﺎﻋﻲ اﻟﻣﺗﺑﻌﮫ ﻟﻠﺣﻣﺎﯾﮫ ﻣن ﻓﺎﯾروس ﻛوروﻧﺎ بشكل دوري؟

نعم لا

1. اعتقد ان اللقاحات المناعية ﻣﮭﻣﮫ ﻟﺻﺣﺔ اﻻطﻔﺎل

نعم لا لا اعلم

1. الﺣﺻول ﻋﻠﻲ اللقاحات ضد الامراض المعدية يقلل من معدل الوفايات و معدل المرضية لدي الافراد

نعم لا لا اعلم

1. عادة مايكون اللقاح ضد الامراض المعدية ذو حماية صحية و يحسن من الكفاءة المعيشية خصوصا عند الأشخاص ذوي المناعة المنخفضة و الذين يعانون من امراض المزمنة

نعم لا لا اعلم

1. ھل ﺗﻌﺗﻘد اﻧﮫ ﻣن اﻟﻣﻣﻛن اﯾﺟﺎد ﻟﻘﺎح ﻓﻌﺎل ﻟﻠﺣﻣﺎﯾﮫ ﻣن ﻓﺎﯾروس ﻛوروﻧﺎ؟

نعم لا

1. في حال إيجاد لقاح فعال, هل تعتقد انه من الممكن ان يتوفر بسهولة لكل الاشخاص؟

نعم لا

1. اضرار اللقاحات عادة تفوق فوائدها الطبية بمراحل

نعم لا لا اعلم

1. ھل ﺗﻌﺗﻘد ﺑﺎن اﻟﻠﻘﺎح ﯾﺟب ان ﯾﻛون ﻣﺟﺎﻧﻲ ﻟﻠﺟﻣﯾﻊ؟

نعم لا

1. اذا توفر اللقاح بمقابل مادي , هل ستقوم بشرائه؟

نعم لا ربما

1. اذا ﻛﺎن ﻟدﯾك اطﻔﺎل, هل سبق وتلقى اي من اطفالك لقاحا يفترض انه يحمي من امراض تحدث خلال فترة الطفولة ؟

نعم لا ليس لدي اطفال

1. ﻓﻲ ﺣﺎل ﺗوﻓر ﻟﻘﺎح ﺑﻔﺎﯾروس ﻛوروﻧﺎ ﺑﻔﺎﻋﻠﯾﺔ 95%, هل ﺳﺗﺳﺗﺧدم ھذا اﻟﻠﻘﺎح ﻟﺗﻔﺎدي اﻻﺻﺎﺑﺔ؟

نعم لا

1. ﻓﻲ ﺣﺎل ﺗوﻓر ﻟﻘﺎح ﺑﻔﺎﯾروس ﻛوروﻧﺎ بفعالية 70% هل ﺳﺗﺳﺗﺧدم ھذا اﻟﻠﻘﺎح ﻟﺗﻔﺎدي اﻻﺻﺎﺑﺔ؟

نعم لا

1. ﻓﻲ ﺣﺎل ﺗوﻓر ﻟﻘﺎح ﺑﻔﺎﯾروس ﻛوروﻧﺎ ﺑﻔﺎﻋﻠﯾﺔ 50%, هل ﺳﺗﺳﺗﺧدم ھذا اﻟﻠﻘﺎح ﻟﺗﻔﺎدي اﻻﺻﺎﺑة؟

نعم لا

1. في ﺣﺎل ﺗوﻓر ﻟﻘﺎح ﺑﻔﺎﯾروس ﻛوروﻧﺎ بالقاعلية المرغوبة, هل ستشجع واﻟدﯾك ﻟﻠﺣﺻول ﻋﻠﻰ اﻟﻠﻘﺎح ﻟﺗﻔﺎدي اﻻﺻﺎﺑﺔ؟

نعم لا

1. ھل ﺣﺻﻠت ﻋﻠﻰ لقاح اﻻﻧﻔﻠوﻧزا اﻟﻣوﺳﻣﯾﺔ ﺧﻼل ﻓﺗرة ال 12ﺷﮭر اﻟﻣﺎﺿﯾﺔ؟

نعم لا

1. ھل ﺗﺳﻌﻰ ﻟﻠﺣﺻول ﻋﻠﻰ لقاح اﻻﻧﻔﻠوﻧزا اﻟﻣوﺳﻣﯾﺔ ﺧﻼل اﻟﺳﻧﺔ اﻟﻘﺎدﻣﺔ

نعم لا
